# Earth-observation variables identify temperature-centered protein-family covariation across macroalgal genomes

**DOI:** 10.1016/j.isci.2026.117777

**Published:** 2026-10-07

**Authors:** Alexandra Mystikou, David R. Nelson, Diana C. El Assal, Ashish K. Jaiswal, Mehar Sultana, Cecilia Rad-Menendez, Noura Al-Mansoori, Sewar T. Elias, Ma-sum Abdul-Hamid, Layanne Nayfeh, Nizar Drou, John A. Burt, David H. Green, Kourosh Salehi-Ashtiani

**Affiliations:** 1 Division of Science, New York University Abu Dhabi, Abu Dhabi, United Arab Emirates; 2 Center for Genomics and Systems Biology (CGSB), New York University Research Institute, New York University Abu Dhabi, Abu Dhabi, United Arab Emirates; 3 Omics Centre of Excellence, M42 Health, Abu Dhabi, United Arab Emirates; 4 Culture Collection of Algae and Protozoa (CCAP), Scottish Association for Marine Science, Oban PA37 1QA, UK; 5 Marine Biodiversity Assessment and Conservation, Terrestrial and Marine Biodiversity, Environment Agency Abu Dhabi, Abu Dhabi, United Arab Emirates; 6 Mubadala Arabian Center for Climate and Environmental Sciences (Mubadala ACCESS), New York University Abu Dhabi, PO Box 129188, Abu Dhabi, United Arab Emirates

**Keywords:** macroalgae, Seaweeds, comparative genomics, Arabian Gulf, vision transformers, earth observation, google earth engine, AlphaEarth Foundations

## Abstract

Macroalgal genome-environment associations are difficult to separate from lineage, geography, and uneven genome quality. We describe nine Arabian Gulf genomes and analyze them with 117 published genomes (126 total). We tested protein family (Pfam) profiles against 13 Google Earth Engine variables. Of 87,123 tests, 84 met global Benjamini-Hochberg q < 0.05, and 49 pairs across 30 Pfams retained selected-family q < 0.05 with discovery-consistent direction in all seven post hoc specifications; all 13 pairs meeting global Bonferroni *p* < 0.05 were retained. The 49 pairs comprised 43 sea surface temperature, five depth, and one distance-to-coast association. PF16087 was positively associated with five temperature summaries, whereas PF12094 and PF01638 were negatively associated with four. A secondary same-site AlphaEarth Foundations crosswalk aligned latent axes A52 and A53 oppositely with five temperature summaries. Together, these analyses place temperature at the center of protein-family covariation in this cohort.

## Highlights

• Earth-observation data link temperature to macroalgal protein-family content
• Forty-nine associations remain supported across seven sensitivity checks
• Forty-three of 49 retained associations involve sea surface temperature
• A learned satellite representation also shows the temperature-centered pattern

## Graphical abstract

**Figure:**
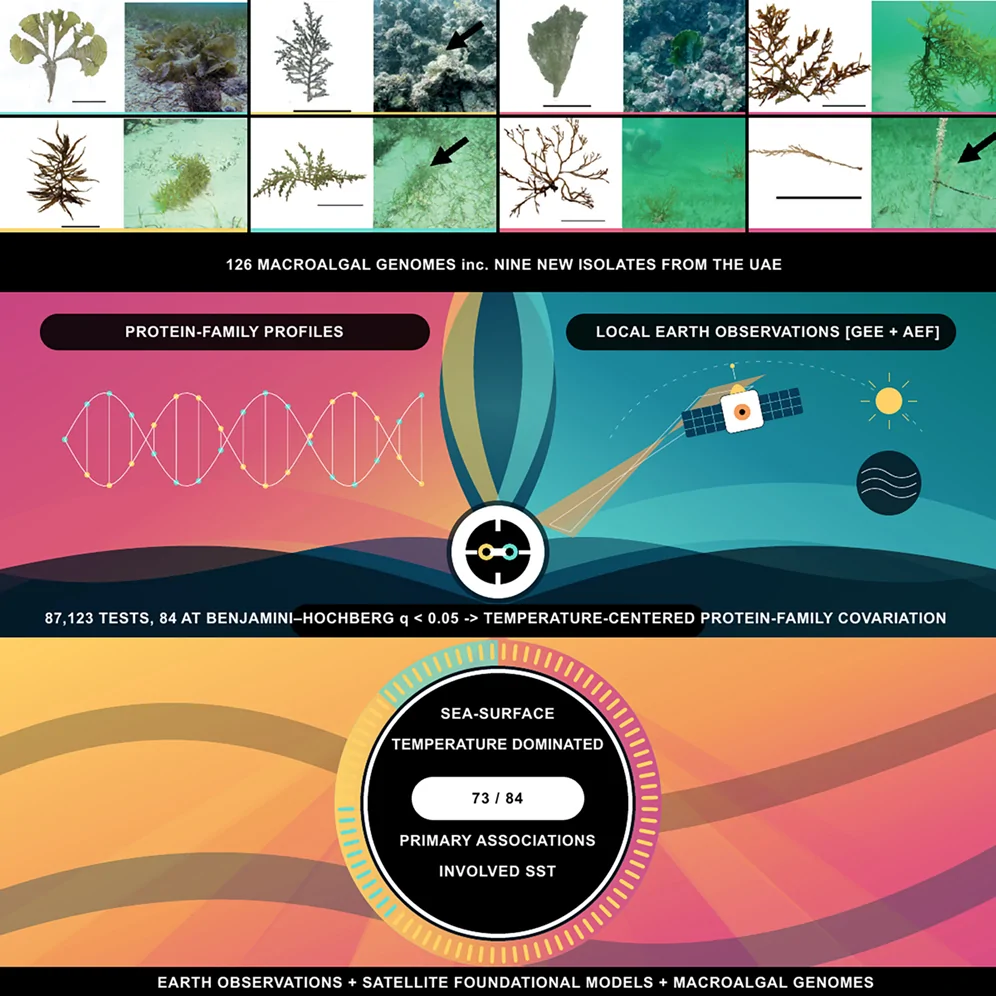

## Teaser

Ecology; Genomics; Bioinformatics

## Introduction

Marine primary producers contribute approximately half of global net primary production, a contribution driven principally by microscopic phytoplankton.^1^ Macroalgae are multicellular photosynthetic organisms broadly classified as brown (Ochrophyta), green (Chlorophyta), and red (Rhodophyta) algae. They span environments from polar waters to tropical reefs and from intertidal habitats to recorded depths exceeding 268 m, where less than 0.1% of surface light penetrates.^2^

As climate change accelerates and human populations grow, seaweed aquaculture is increasingly recognized as a sustainable source of food and natural products and as a potential nature-based option for climate change mitigation and adaptation.^3^ The Kelp Forest Challenge exemplifies a large-scale international initiative to protect and restore kelp habitats.^4^ At the same time, macroalgal range expansions create monitoring challenges. The Mediterranean spread of *Caulerpa taxifolia* var. *distichophylla*^5^ and environmental-DNA detection difficulties for invasive *Caulerpa prolifera*^6^ reveal taxon-specific monitoring blind spots; the Pacific-to-Atlantic spread of *Gracilaria vermiculophylla*^7^ and transcontinental establishment of *Undaria pinnatifida*^8^ show how widely introduced macroalgae can establish.^9^ Yet our understanding of macroalgal genomics lags far behind this ecological breadth. Genome-scale studies encompass only a small fraction of species,^10^^,^^11^^,^^12^^,^^13^^,^^14^^,^^15^^,^^16^^,^^17^^,^^18^^,^^19^^,^^20^ although more than 10,000 macroalgal species have been described. Available genomes are also unevenly distributed among lineages and locations, differ in quality, and sometimes lack complete collection metadata. These gaps make the genomic correlates of environmental distributions difficult to resolve,^21^ constraining evidence-based interpretation of climate responses and biotechnological traits.

The Arabian Gulf brings this challenge into sharp focus. As one of the world’s most thermally and osmotically extreme marine environments, it experiences summer temperatures exceeding 35°C^22^ and salinities exceeding 44 practical salinity units (PSU)^23^^,^^24^^,^^25^^,^^26^^,^^27^^,^^28^—conditions that push marine life toward physiological limits and may represent future ocean states elsewhere under climate change.^29^^,^^30^^,^^31^ The Arabian Gulf genomes therefore broaden the environmental range represented in comparative macroalgal genomics.

We therefore describe and analyze nine macroalgal genomes from the United Arab Emirates (UAE) alongside 117 published genomes (Table S1). Their draft assemblies were previously deposited and included as public data in a large-scale comparative genomics study,^32^ but their isolation, environmental context, and genomic characterization had not been formally described.

We used a primary named-variable analysis and a secondary learned-representation analysis to connect genome content with environmental context. Google Earth Engine (GEE)^33^ supplied 13 named oceanographic and geospatial variables, whereas the 64-dimensional AlphaEarth Foundations (AEF) product^34^^,^^35^ supplied a jointly learned representation of the same recorded coordinates. Each genome identifier (ID) was matched one-to-one across protein family (Pfam)^36^ profiles, coordinates, and environmental features. All 84 GEE discovery pairs meeting global Benjamini-Hochberg (BH)^37^ q < 0.05 entered the seven-specification post hoc sensitivity family. Separately, four priority Pfam-AEF pairs and PF00092 across all 64 axes formed the 68-pair AEF sensitivity family. A unique-site AEF-GEE alignment then provided a site-level cross-representation crosswalk for the latent axes. This design separates primary named-variable associations from secondary same-site cross-representation context while retaining an observational scope.

## Results

### The 126-genome cohort spans major macroalgal lineages and climate zones

The 126-genome cohort included 70 Rhodophyta, 43 Ochrophyta, and 13 Chlorophyta from 77 genera (Figure 1; Table S1F). It spanned recorded Polar (*n* = 22), Subpolar (*n* = 2), Temperate (*n* = 52), Subtropical (*n* = 27), and Tropical (*n* = 23) settings, broadening representation of environments that remain sparsely sampled in macroalgal genomics. Figure 2 places this cohort in phylogenetic context by showing the 96 species-resolved tips with complete, concordant recorded metadata from the deposited 119-tip RbcL tree. This display subset does not alter the 126-genome cohort or the 112-genome topology sensitivity.

**Figure 1:**
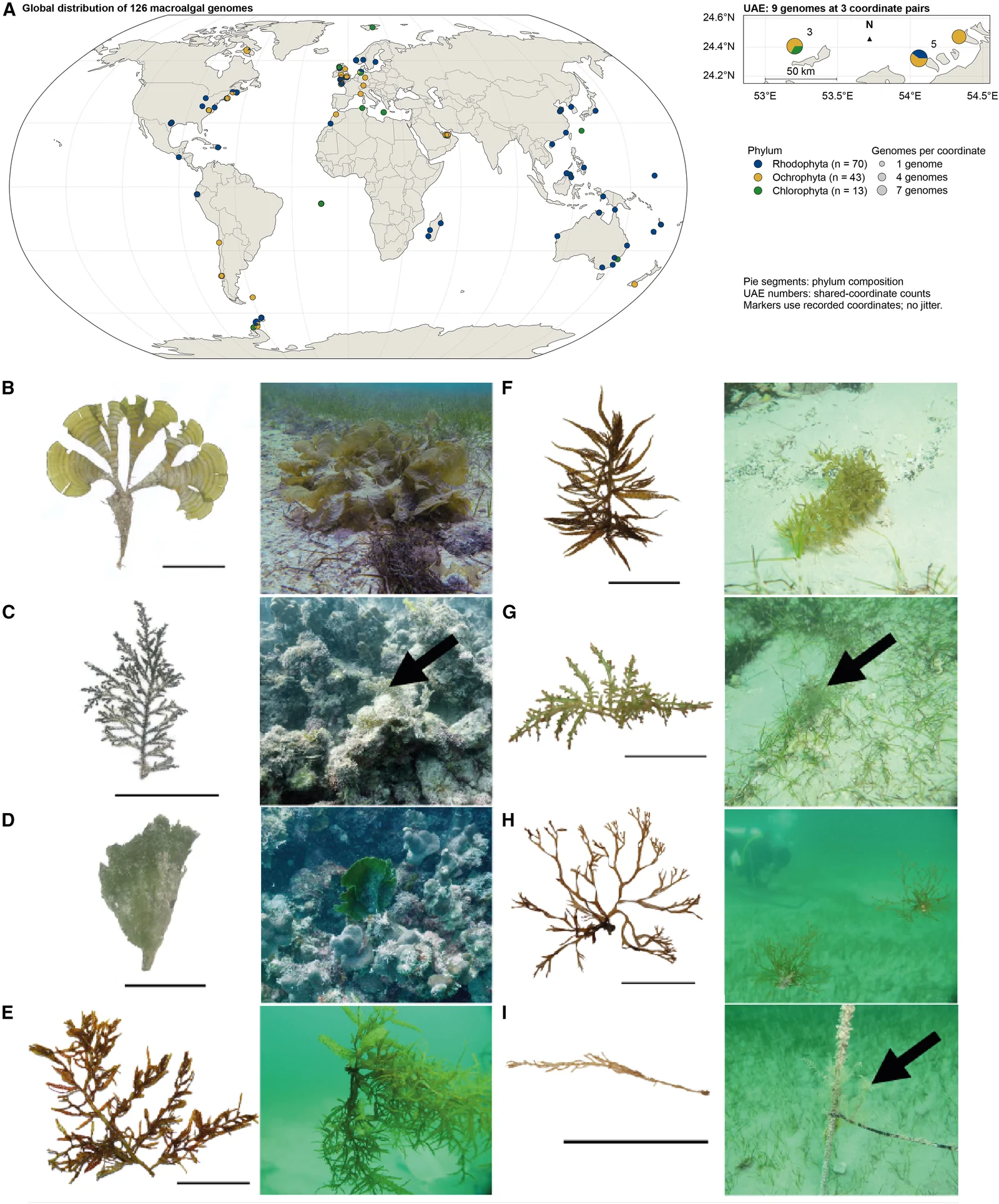
Geographic distribution of macroalgal genomes and representative Arabian Gulf specimens (A) Global distribution of 126 macroalgal genomes at 90 recorded coordinate pairs. Pie-marker area represents the number of genomes at each coordinate, and segments show phylum composition. Nineteen coordinate pairs are shared by multiple genomes (maximum, seven genomes per coordinate). The inset shows the nine United Arab Emirates (UAE) genomes at three coordinate pairs; markers use the exact recorded coordinates. The inset scale bar represents 50 km. (B–I) Eight specimen panels represent the nine UAE genomes because panel D includes the photographed chlorophyte host and its endophytic brown-algal genome. Left image show herbarium specimens; right image show *in situ* underwater photographs demonstrating natural habitat and growth forms. Scale bars, 5 cm. Species shown: (B) *Padina boergesenii* (NYAE_20191204_1), Ochrophyta. The rolled-blade morphology is typical of Dictyotales. (C) *Polycladia myrica* (NYAE_20191205_1), Ochrophyta. Black arrow indicates pneumatocysts (gas-filled flotation structures). (D) *Avrainvillea amadelpha* (NYAE_20191205_6a), Chlorophyta. This specimen hosted an endophytic brown alga (NYAE_20191205_6b). (E) *Sargassum latifolium* (NYAE_20200302_1), Ochrophyta. Representative of the dominant macroalgal genus in the Arabian Gulf. (F) *Sargassum angustifolium* (NYAE_20200302_2), Ochrophyta. Black arrow indicates characteristic narrow blade morphology distinguishing it from *S. latifolium*. (G) *Chondria dasyphylla* (NYAE_20200302_4), Rhodophyta. The panel shows the branching thallus morphology. (H) *Canistrocarpus cervicornis* (NYAE_20200302_5), Ochrophyta. The specimen was epizoic on a gastropod shell. (I) Specimen identity and locality are unresolved in the collection records. Collection metadata are provided in Table S1A; the 126 genomes and recorded coordinate pairs shown in A are listed in Table S1F.

**Figure 2:**
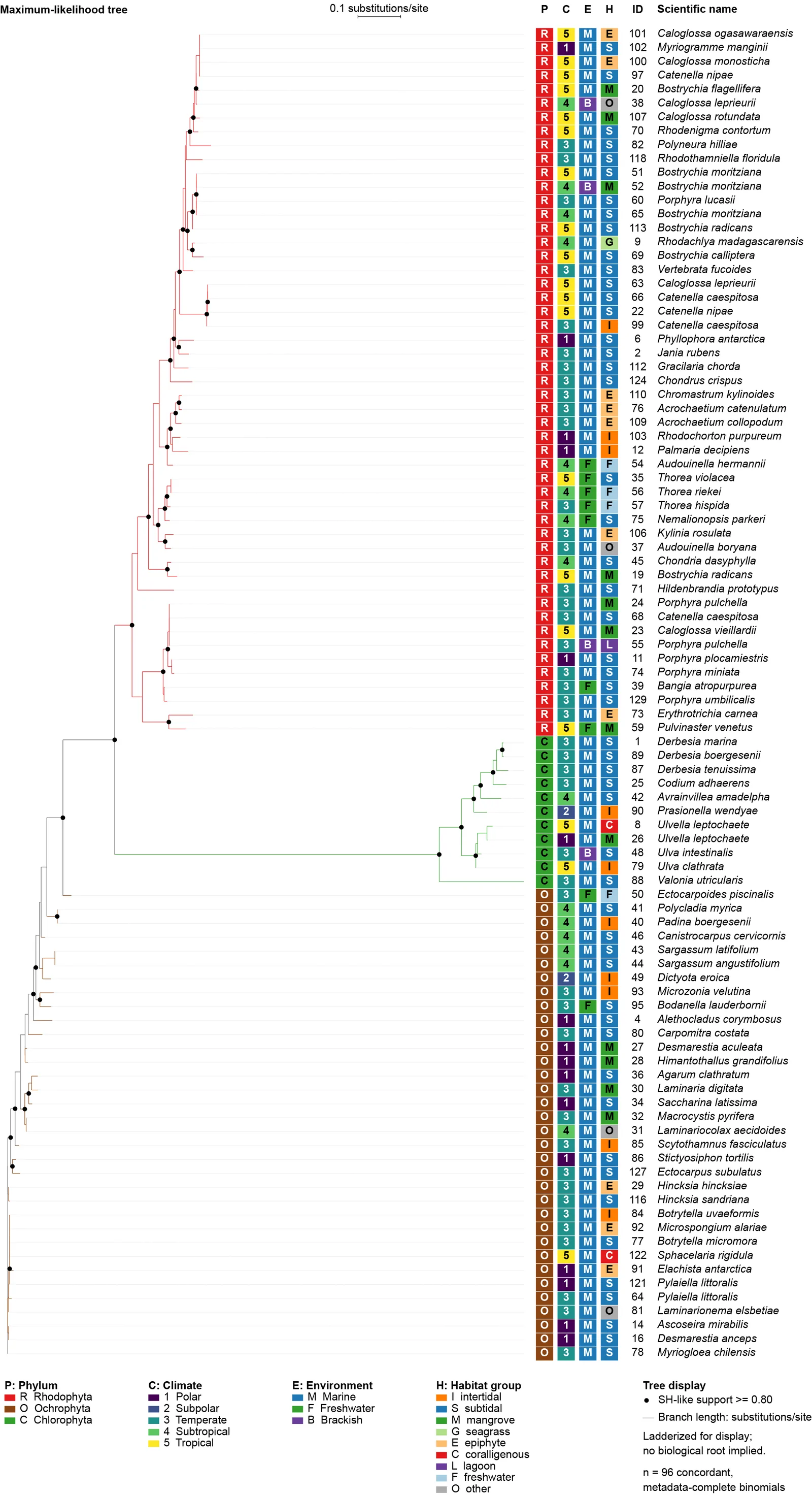
Macroalgal RbcL phylogeny and recorded metadata Display-filtered maximum-likelihood phylogeny derived from the deposited 119-tip ribulose-1,5-bisphosphate carboxylase/oxygenase large-subunit (RbcL) protein tree, which was inferred with FastTree 2.1.11^38^ under the Jones-Taylor-Thornton amino acid substitution model with a 20-category approximation of among-site rate variation (JTT+CAT20) and pruned only for presentation. Tips were retained only when accession-gated reconciliation yielded a unique authoritative metadata record with every displayed field, a species-level scientific name, and no unresolved cross-source identity or phylum conflict. The display retains 96 tips; 23 were omitted, comprising 12 genus-only names, three without a safe metadata mapping, and eight with unresolved cross-source conflicts. The displayed set contains 92 members of the 126-genome analysis cohort and four authoritative-inventory-only records and therefore is not itself an analytical cohort. The 96 tips comprise 51 Rhodophyta, 34 Ochrophyta, and 11 Chlorophyta. The induced topology and support values were inherited from the full-tree inference and were not recomputed; pruning preserved pairwise patristic distances among retained tips. Terminal order was ladderized for display without rerooting, and no biological root is implied. Filled circles mark Shimodaira-Hasegawa-like local support ≥0.80. The scale bar indicates branch length in substitutions per site. Side tracks give P, phylum; C, climatic zone; E, environment; and H, habitat group, with character codes duplicating color encodings. Tip IDs link records to Table S1C.

The nine Arabian Gulf genomes carry this cohort into hypersaline, hyperthermal conditions relevant to climate comparisons.^39^ They span brown, red, and green algal lineages, represented by the specimens in Figure 1, and derive from three exact coordinate pairs. Several were collected from recently bleached coral substrates, while *Avrainvillea amadelpha* has also been reported as a potential Mediterranean invader.^40^ Where specimen identity, locality, or depth records conflict, we report those fields as unresolved rather than inferring a value.

### Named environmental variables identify temperature-centered Pfam covariation

To test whether Pfam abundance covaried with named environmental conditions, we joined reconstructed raw hidden Markov model (HMM) counts to 13 GEE variables by canonical genome ID. Coverage ranged from 72 to 126 genomes per variable and represented up to 90 exact coordinate sites. A parallel screen of recorded metadata, including temperature, latitude, longitude, and environment category, is reported separately in Figures 3 and 4.

**Figure 3:**
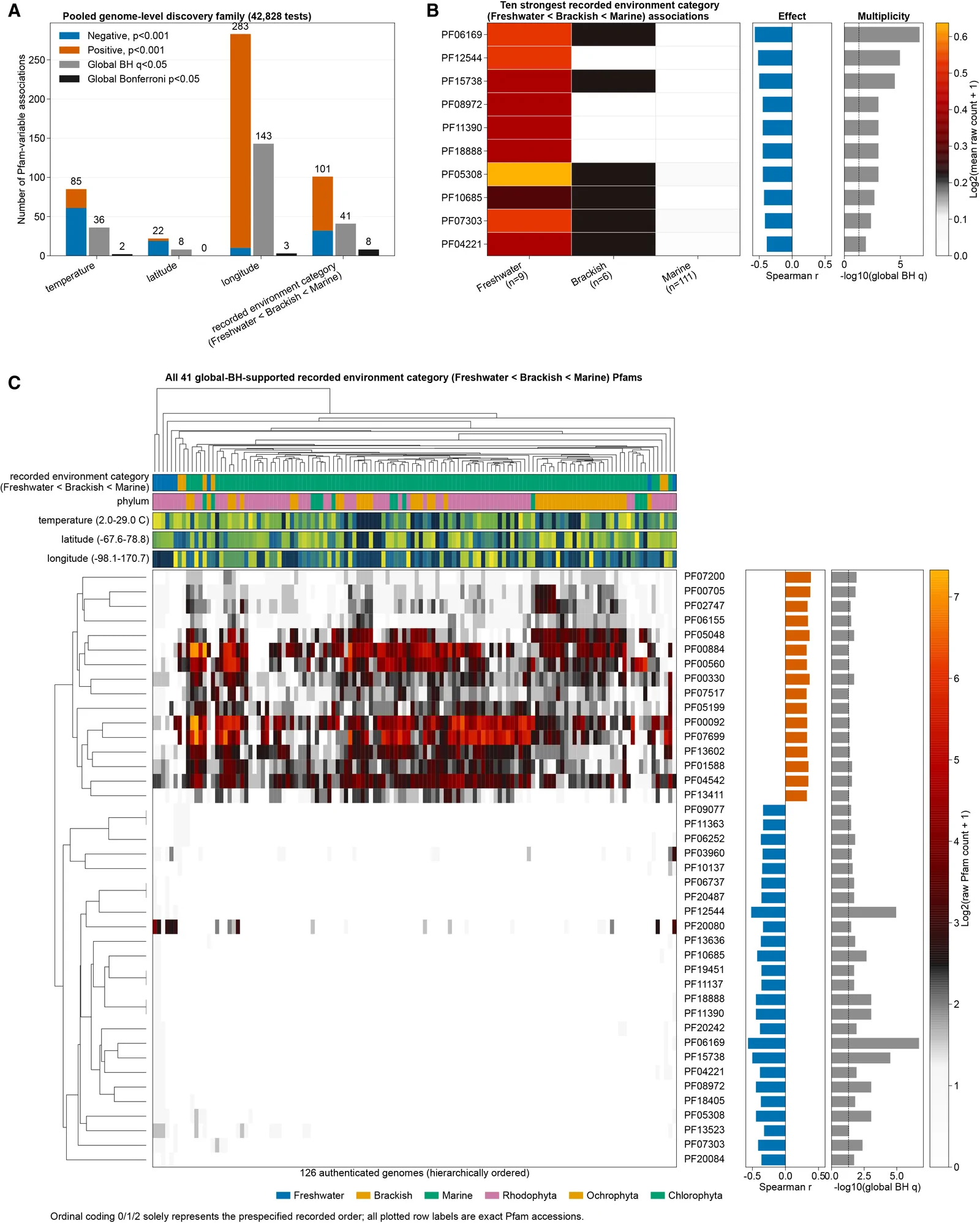
Recorded metadata-protein family discovery analysis in the 126-genome cohort (A) Counts of Pfam associations with recorded temperature, latitude, longitude, and the recorded environment category ordered Freshwater < Brackish < Marine. Bars report negative and positive associations at *p* < 0.001, global Benjamini-Hochberg q < 0.05, and global Bonferroni *p* < 0.05 across the 42,828-pair family. The recorded environment category used the prespecified ordinal coding Freshwater = 0, Brackish = 1, and Marine = 2. (B) The ten smallest raw *p* value associations with the recorded environment category. The heatmap shows log2(mean raw count +1) for Freshwater, Brackish, and Marine genomes; side images show Spearman r and −log10(global Benjamini-Hochberg q). (C) Raw log2(count +1) profiles for all 41 Pfams associated with the recorded environment category at global q < 0.05 across 126 genomes. Tracks show recorded environment category, phylum, temperature, latitude, and longitude; side panels show Spearman r and −log10(global q). Average-linkage Euclidean clustering organizes the complete 41-Pfam × 126-genome row-*Z*-scored matrix.

**Figure 4:**
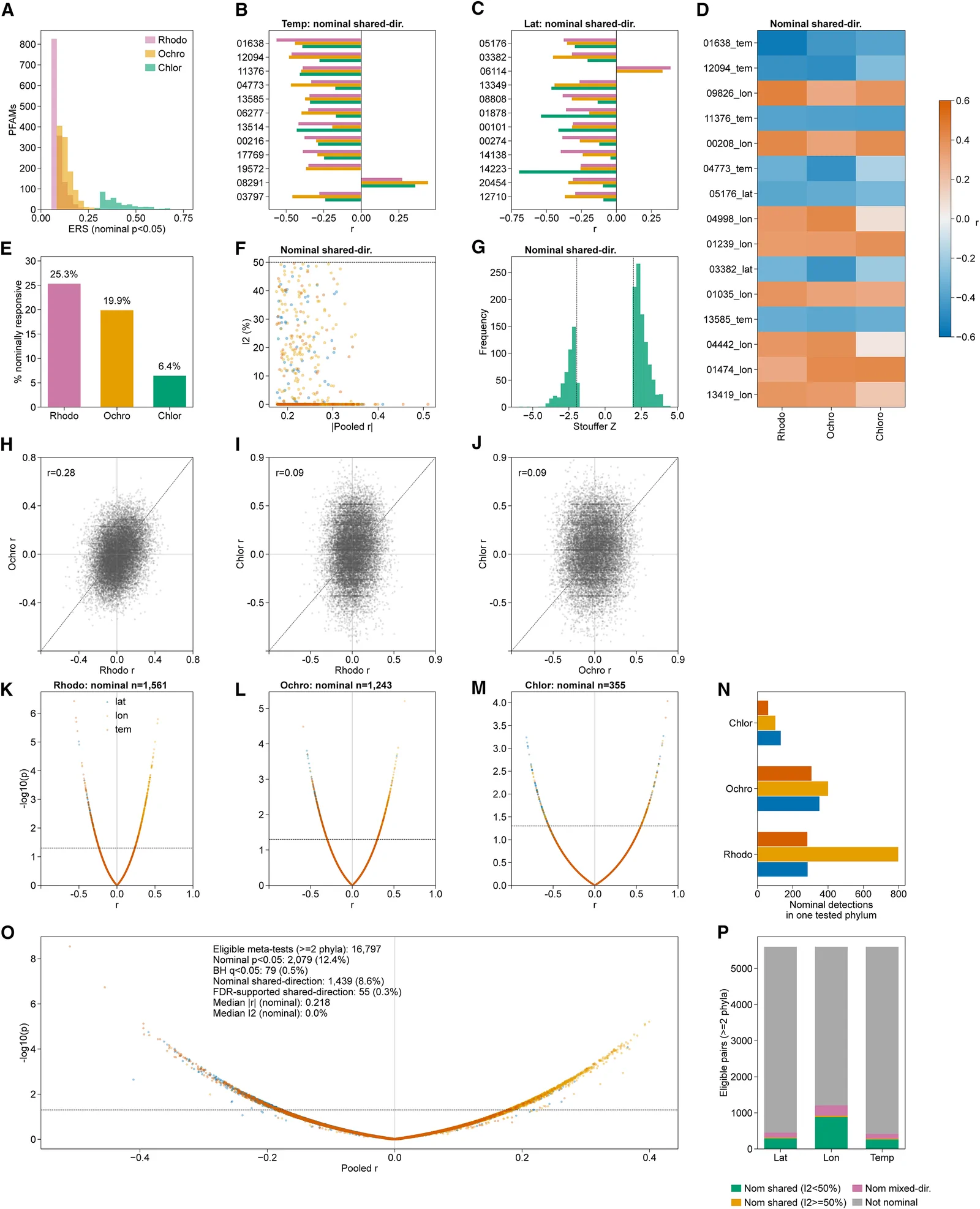
Within-phylum protein family associations with recorded metadata in the 126-genome cohort The analysis used recorded temperature, latitude, and longitude for 70 Rhodophyta, 43 Ochrophyta, and 13 Chlorophyta. (A–P) show within-phylum correlation, concordance, nominal-screening, phylum-specific, weighted signed Stouffer, and heterogeneity summaries. Across 16,797 eligible meta-analysis tests, 79 passed Benjamini-Hochberg q < 0.05 and 55 had a shared effect direction with I^2^ < 50%. In the images, environmental response strength (ERS) is the mean of the squared Spearman coefficients (r_s_^2^) across recorded-environment tests with nominal *p* < 0.05 for a Pfam within a phylum, with zero assigned when no test meets that threshold. BH denotes Benjamini-Hochberg correction, FDR denotes false discovery rate, I^2^ denotes the I-squared heterogeneity statistic, r denotes the Spearman correlation coefficient, and *Z* denotes the Stouffer statistic. Pfam labels denote accession identifiers. Rhodo, Ochro, Chlor, and Chloro denote Rhodophyta, Ochrophyta, and Chlorophyta; Temp/tem, Lat/lat, and Lon/lon denote temperature, latitude, and longitude; Nom denotes nominal; and shared-dir. and mixed-dir. denote shared direction and mixed direction.

After the 5% prevalence filter, 6,700–6,703 Pfams were estimable per variable, yielding 87,123 Spearman tests. Sixteen of 87,139 possible Pfam-variable combinations were nonestimable because the Pfam vector was constant within the relevant complete-case subset (Tables S2B and S2C). Of the estimable tests, 84 met global Benjamini-Hochberg q < 0.05 and 13 met global Bonferroni *p* < 0.05, spanning 51 Pfams. Sea surface temperature (SST) accounted for 73 global-BH associations, depth for 10, and distance to coast for one; the other named variables contributed none at this threshold.

Temperature dominated the discovery pattern. PF16087 (DUF4817; helix-turn-helix domain [DUF4817]) was positively associated with mean, maximum, minimum, summer, and winter SST (r = 0.435 to 0.579; global q = 5.49 × 10^−6^ to 0.0454). PF12094 (DUF3570; protein of unknown function [DUF3570]) was negatively associated with maximum, mean, minimum, and winter SST (r = −0.533 to −0.489; q = 6.07 × 10^−5^ to 0.00337). PF01638 (HxlR; HxlR-like helix-turn-helix) showed negative associations with the same four summaries (r = −0.518 to −0.497; q = 1.41 × 10^−4^ to 0.0011).

Sensitivity analyses narrowed the 84 global-BH discoveries to a stable subset (Figure 5; Table 1; Tables S2C and S2E–S2G). All 84 pairs entered seven required specifications, and 56 met selected-family q < 0.05 in the structured-null specification. Forty-nine pairs across 30 Pfams retained selected-family q < 0.05 with discovery-consistent direction in all seven specifications. This set comprised 43 of 73 SST pairs, 5 of 10 depth pairs, and the single distance-to-coast pair. All 13 global-Bonferroni discovery pairs retained support in all seven specifications.

**Figure 5:**
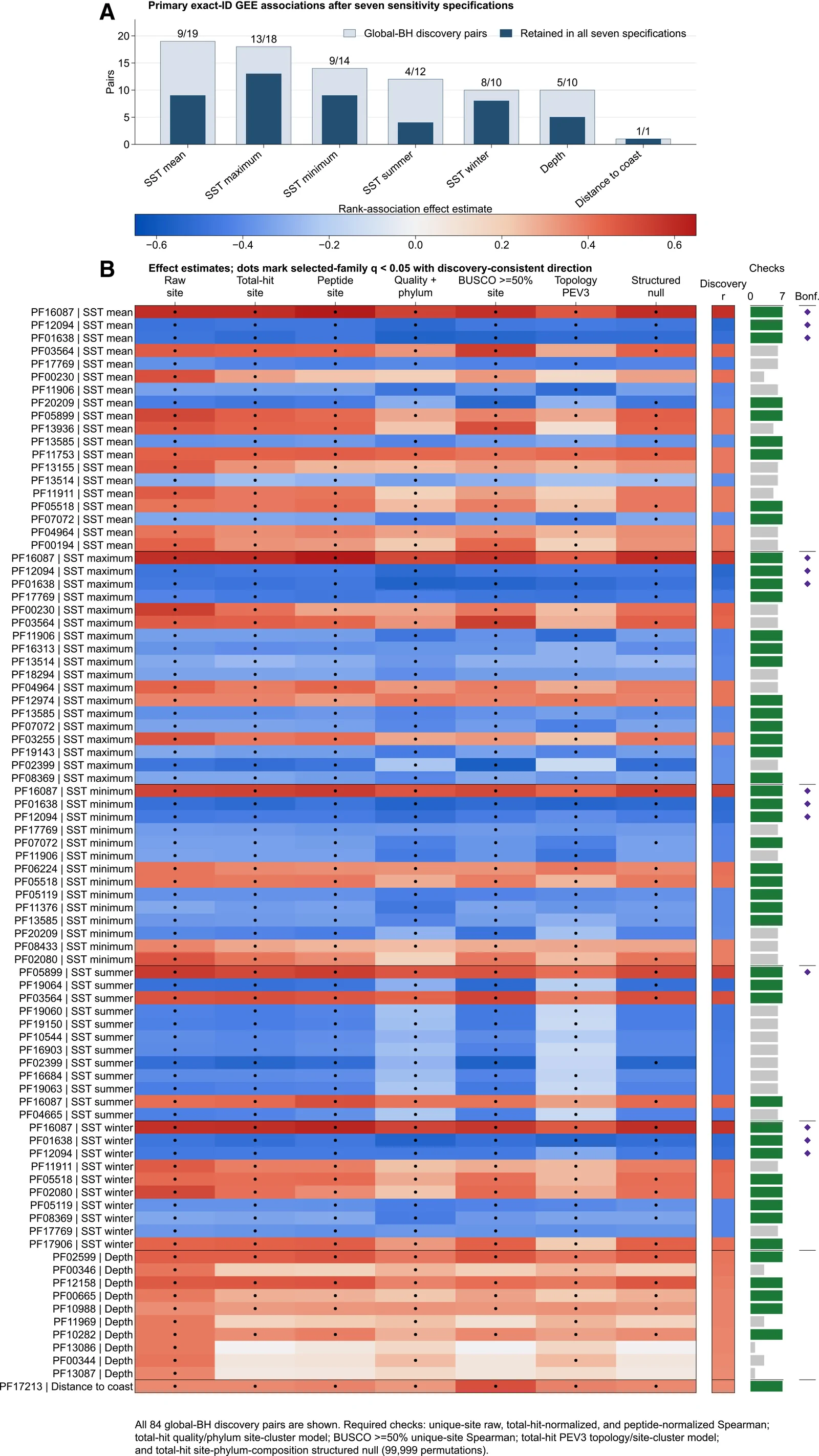
Primary exact genome identifier Google Earth Engine associations across seven sensitivity specifications (A) Counts of the 84 global Benjamini-Hochberg (BH) discovery pairs and the 49 pairs retaining selected-family support with consistent direction in all seven required specifications, grouped by named GEE variable. (B) Effect estimates for every selected pair across raw-count unique-site, total-hit-normalized unique-site, peptide-normalized unique-site, quality/phylum coordinate-clustered, Benchmarking Universal Single-Copy Orthologs (BUSCO) ≥50% unique-site, three-eigenvector topology coordinate-clustered, and site phylum-composition structured-null specifications. Dots mark selected-family q < 0.05 with discovery-consistent direction. The right tracks show the discovery effect, number of passing specifications, and the 13 global-Bonferroni discovery pairs; all 13 passed all seven required specifications. PEV denotes phylogenetic eigenvector.

**Table 1:** Recurring sea surface temperature-associated protein families within the 49-pair primary exact genome identifier Google Earth Engine result set

Pfam (verified label)	Named GEE variables	Discovery r	Global BH q	Unique-site r	Retention (all-seven; Bonf.)
PF16087 (DUF4817)	SST mean, maximum, minimum, summer, winter	0.435 to 0.579	5.49 × 10^−6^ to 0.0454	0.413 to 0.590	5/5; 4
PF12094 (DUF3570)	SST mean, maximum, minimum, winter	−0.533 to −0.489	6.07 × 10^−5^ to 0.00337	−0.481 to −0.453	4/4; 4
PF01638 (HxlR)	SST mean, maximum, minimum, winter	−0.518 to −0.497	1.41 × 10^−4^ to 0.0011	−0.489 to −0.461	4/4; 4
PF05899 (EutQ-like cupin domain)	SST mean, summer	0.402 to 0.527	0.00228 to 0.036	0.519 to 0.554	2/2; 1

The primary retained set contains 49 pairs across 30 Pfams. The four recurring accessions shown here account for all 13 pairs meeting global Bonferroni *p* < 0.05 and 15 of the 49 retained pairs meeting global Benjamini-Hochberg (BH) q < 0.05; labels are verified accession-level Pfam/InterPro names. Discovery and unique-site ranges cover every selected SST association for each accession. The retention column reports all-seven pairs followed by the number meeting the global Bonferroni threshold. Full annotated results are reported in Table S2.

The global-Bonferroni pairs showed the same temperature-centered pattern. PF16087 had four positive SST associations (discovery r = 0.538 to 0.579; unique-site r = 0.526 to 0.590; topology-adjusted effect = 0.436 to 0.467). PF12094 had four negative associations (discovery r = −0.533 to −0.489; unique-site r = −0.481 to −0.453; topology-adjusted effect = −0.457 to −0.314), and PF01638 had four negative associations (discovery r = −0.518 to −0.497; unique-site r = −0.489 to −0.461; topology-adjusted effect = −0.539 to −0.507). PF05899-summer SST also retained support (discovery r = 0.527; unique-site r = 0.554; topology-adjusted effect = 0.415).

Accession-level InterPro^41^ annotations placed the retained Pfams in several broad classes rather than a single functional category. The SST-associated set included carboxyltransferase-like (PF03255), phosphonate-binding ABC-transporter (PF12974), DNA glycosylase AlkZ-like (PF06224), proto-chlorophyllide reductase (PF08369), and EutQ-like cupin (PF05899) labels; regulatory or helix-turn-helix labels included PF16087 and PF01638, while PF02599 was depth-associated. Mobile-element, viral, and unknown-function labels were also represented, including eight accessions with unknown-function labels. These database annotations do not establish protein function in macroalgae.

Changing the temperature product preserved effect direction but not the complete BH-selected membership. A post hoc product sensitivity used the Hybrid Coordinate Ocean Model (HYCOM)^42^^,^^43^ as a comparator to the Moderate Resolution Imaging Spectroradiometer (MODIS) Aqua product.^44^^,^^45^ Across six temperature summaries, MODIS-HYCOM Spearman correlations were ρ = 0.901–0.993 at exact sites and ρ = 0.930–0.995 after equal-weight native-cell aggregation (Tables S2J and S2L). HYCOM matched the MODIS coefficient direction for all 73 selected SST-Pfam pairs at both levels and yielded 42 pairs with BH q < 0.05 in each analysis, although membership differed (Tables S2I–S2M).

### Recorded metadata also identify the principal temperature-associated Pfams

A separate screen tested 10,707 strict Pfams against World Ocean Atlas 2018 (WOA2018) recorded temperature,^46^ latitude, longitude, and the ordinal environment category Freshwater < Brackish < Marine in the same 126-genome cohort (Figure 3). Among 42,828 tests, 491 had *p* < 0.001, 228 met global Benjamini-Hochberg q < 0.05, and 13 met global Bonferroni *p* < 0.05. The global-BH set comprised 143 longitude, 36 temperature, 8 latitude, and 41 recorded-environment-category associations.

The supporting metadata screen also identified two Pfams at the center of the primary temperature pattern. PF01638 had the strongest recorded-temperature association (r = −0.480, *p* = 1.27 × 10^−8^, global q = 1.36 × 10^−4^), and PF12094 was also negatively temperature-associated (r = −0.442, *p* = 2.19 × 10^−7^, global q = 1.04 × 10^−3^). The strongest association overall linked PF06169 to recorded environment category (Spearman r = −0.567, *p* = 4.55 × 10^−12^, global q = 1.95 × 10^−7^). The 13 global-Bonferroni associations comprised 8 recorded-environment-category, 3 longitude, 2 temperature, and 0 latitude pairs.

Within-phylum analysis further separated consistent from heterogeneous associations (Figure 4). Across 16,797 eligible meta-analyses represented by at least two phyla, 2,079 had nominal weighted Stouffer^47^ *p* < 0.05 and 79 met Benjamini-Hochberg q < 0.05. Fifty-five of those 79 shared an effect direction and had an I-squared (I^2^) heterogeneity statistic^48^ below 50%.

### PF00092 provides a contrasting result without consistent UAE enrichment

PF00092 domains showed heterogeneous metal-ion-dependent adhesion site (MIDAS) signatures across the cohort. The von Willebrand factor type-A (vWF-A) domain (PF00092) is a conserved protein-interaction module, and structurally characterized vWF-A domains differ in MIDAS architecture.^49^^,^^50^^,^^51^^,^^52^ We recovered exact peptide sequences for 2,024 of 2,128 original PF00092.31 protein-hit records and reannotated them with the pinned PF00092.35 profile, retaining 1,968 proteins containing 2,160 domains. Of the 1,595 domains aligned across all five canonical MIDAS positions (D-X-S-X-S … T … D), 549 domains in 536 proteins carried the complete canonical signature across 94 genomes, whereas 1,046 were noncanonical; the remaining 565 alignments were incomplete at one or more positions.

At the genome level, mean reconstructed raw HMM counts were 1.73-fold higher in the nine UAE genomes than in the 117 comparison genomes (27.8 versus 16.1 hits). Because those nine genomes represented only three of the 90 coordinate sites, we next aggregated the counts by site. The UAE-to-non-UAE ratio then fell to 1.261 (95% bootstrap confidence interval [CI], 0.371–2.564; *p* = 0.386; three versus 87 sites). Ratios based on total Pfam hits and peptide records were 1.345 (95% CI, 0.340–2.414; *p* = 0.514) and 2.467 (95% CI, 0.097–5.880; *p* = 0.576; three versus 82 sites), respectively. Thus, the apparent contrast depended on analysis scale and denominator.

Additional post hoc estimates remained inconsistent. For total-hit-normalized abundance, the structured site-label permutation yielded *p* = 0.264, and the primary topology-aware coefficient was β = 0.123 (95% CI, −0.032–0.278; *p* = 0.120; 112 genomes at 82 sites). The coordinate-clustered quality model yielded β = 0.159 (95% CI, 0.015–0.302; *p* = 0.0298). Taken together, the UAE comparisons did not consistently support higher PF00092 abundance.

### The same-site AEF analysis aligns with the temperature-centered pattern

We next returned to the cohort-wide temperature pattern by asking whether it was also represented in a learned description of the same locations. We analyzed the precomputed 64-dimensional AlphaEarth Foundations (AEF) product. Related machine-learning and multi-source remote-sensing approaches have been used in multi-environment genomic prediction,^53^ protein-interaction screening,^54^ and biodiversity assessment.^55^ We sampled GOOGLE/SATELLITE_EMBEDDING/V1/ANNUAL as documented by its primary source^34^ and data catalog.^35^ Because the 64 axes are unitless, jointly learned features, we interpreted them through covariance with named GEE variables rather than as physical environmental measurements.

The pooled screen evaluated 685,248 Pfam-AEF pairs and identified 24 pairs spanning 17 Pfams and 13 AEF axes at global Benjamini-Hochberg q < 0.05, including three pairs at global Bonferroni *p* < 0.05 (Table S3A). The phylum-centered screen identified 17 pairs at global q < 0.05 (Table S3B). Average-linkage clustering^56^ of all 10,707 row-wise *Z*-scored 64-axis profiles retained every accession in Figure S2 and resolved 8,713 unique profiles plus 1,994 accessions sharing an exact profile.

Four pooled pairs defined the priority AEF subset: PF13411-A18 (r = 0.428, global q = 3.56 × 10^−2^), PF01638–A52 (r = 0.456, global q = 1.27 × 10^−2^), PF01638-A53 (r = −0.487, global q = 2.52 × 10^−3^), and PF10988-A36 (r = 0.443, global q = 2.1 × 10^−2^; Table S3A). HMM and InterPro^41^ records annotate PF13411 as MerR_1 (MerR helix-turn-helix family regulatory protein), PF01638 as HxlR (HxlR-like helix-turn-helix), and PF10988 as DUF2807 (putative auto-transporter adhesin, head GIN domain). These labels provide sequence context only; their AEF relationships remain latent-feature covariance.

No within-phylum Pfam-AEF pair survived global Benjamini-Hochberg correction. Screens requiring at least three nonzero genomes evaluated 4,761 Pfams in Chlorophyta, 5,856 in Ochrophyta, and 7,173 in Rhodophyta and yielded 245, 832, and 1,007 pairs with *p* < 0.001, respectively, but zero pairs at global q < 0.05 in each phylum (Table S3F). A Rhodophyta sensitivity requiring at least ten nonzero genomes evaluated 5,130 Pfams (328,320 pairs), identified 905 pairs with *p* < 0.001, and again yielded zero pairs at global q < 0.05 (minimum q = 0.0655; Table S3F).

PF01638-A52 and PF01638-A53 were the only priority AEF pairs that retained support across all seven specifications (Figure S1; Table S3). At 90 unique sites, their raw-count effects were ρ = 0.428 and −0.508, respectively. For total-hit-normalized abundance, the corrected structured-null results were A52 ρ = 0.450, *p* = 0.00008, selected-family q = 0.00272; and A53 ρ = −0.542, *p* = 0.00002, q = 0.00136. PF10988-A36 and PF13411-A18 each retained support in five of seven specifications. Among the 64 PF00092-axis pairs, none retained support across all seven specifications; PF00092-A49 passed only the raw-count unique-site specification (Table S3).

The site-level AEF-GEE crosswalk connected A52 and A53 to the same named SST summaries associated with PF01638. Across 832 correlations at 48–90 sites, 224 met global BH q < 0.05 and 44 met global Bonferroni *p* < 0.05 (Table S3). A52 aligned negatively and A53 positively with GEE mean, minimum, maximum, summer, and winter SST across 57–72 sites (|ρ| = 0.454–0.704; global q ≤ 0.00404). Because PF01638 was positively associated with A52 and negatively associated with A53, the sign products agreed with the four direct negative PF01638-GEE associations shared by the analyses (mean, minimum, maximum, and winter SST). This same-site cross-representation alignment does not assign a unique meaning to either latent axis.

## Discussion

### Temperature-centered Pfam covariance persists across analyses

Temperature was the dominant environmental dimension associated with protein-family content in this cohort. SST accounted for 73 of 84 discovery pairs and 43 of 49 pairs that retained selected-family support with consistent direction across all seven specifications. PF16087 showed recurring positive SST associations, whereas PF12094 and PF01638 showed recurring negative associations. The supporting recorded-metadata analysis also identified negative temperature associations for PF12094 and PF01638, and substituting HYCOM for MODIS preserved the direction of all 73 selected SST-Pfam coefficients at covered sites, although BH-selected membership differed. Thus, neither the tested product substitution nor the stated sensitivity analyses reversed the temperature-centered pattern.

The AEF analysis provided a second representation of the same temperature-centered pattern. A52 and A53 aligned in opposite directions with five named SST summaries and with PF01638, yielding sign agreement with the four direct PF01638-SST associations shared by the analyses. Because both representations were sampled at the same coordinates, this concordance is same-site cross-representation consistency rather than independent validation; the latent axes remain distributed and nonunique.

PF00092 provided a contrasting result. Although genome-level raw counts were 1.73-fold higher in the nine UAE genomes, the site-level and normalized estimates were imprecise, and support varied across specifications. The canonical MIDAS signatures cataloged across the cohort describe sequence composition; they do not establish higher PF00092 abundance in UAE genomes or attachment-related function.

### Accession-level labels contextualize the sequence families

The retained associations spanned broad annotation categories rather than a single mechanistic class. InterPro^41^ labels included regulatory and helix-turn-helix proteins, carboxyltransferase-like and phosphonate-binding proteins, DNA glycosylase AlkZ-like proteins, proto-chlorophyllide reductase, mobile-element and viral families, and proteins of unknown function. These accession-level labels contextualize the sequence families but do not establish their roles in macroalgae.

Climate-related biological responses are documented across oceans,^57^ and adaptation from standing genetic variation provides a general evolutionary framework.^58^ However, this cohort-level analysis does not test population-level adaptation or selection; it measures Pfam-environment covariance within a cohort structured by lineage and geography.

Genomic diversity is a recognized conservation goal.^59^ Extreme Arabian Gulf conditions and broader marine-restoration priorities provide ecological context for why these genomic resources matter.^60^^,^^61^ Within the evidence reported here, however, the contribution is narrower: temperature-centered Pfam covariance recurred across named variables, the stated sensitivity analyses, and a learned representation of the same sites.

### Limitations of the study

The cohort’s breadth is accompanied by uneven sampling. The 126 genomes represent 90 exact coordinate sites, but the nine UAE genomes come from only three sites, and phyla are unevenly represented. Public records rarely include quantitative positional uncertainty, so the analysis necessarily used recorded points rather than data-driven buffers or exclusions. The named GEE summaries describe 2020–2023 conditions with variable-specific coverage of 72–126 genomes. The archived AEF extractor mosaicked the unfiltered annual ImageCollection, with overlap priority following collection order; the site-level crosswalk therefore compares coordinate-linked representations with different temporal provenance.

The sensitivity analyses address several dependencies but cannot remove them. Unique-site estimates and coordinate-clustered models address repeated locations; quality covariates and benchmarking universal single-copy orthologs (BUSCO)^62^ subsets address assembly quality; alternative denominators change the estimated abundance quantity; and topology eigenvectors address accession-gated relatedness. The structured null preserves intact sites within site phylum-composition strata but not geographic distances or spatial autocorrelation among distinct sites. Because the seven specifications are correlated reanalyzes and selected-family q-values apply only to the stated 84-pair GEE or 68-pair AEF families, agreement indicates stability to those choices rather than seven independent validations.

Raw counts and normalized ratios measure different genomic quantities, as PF00092 illustrates. The AEF-GEE crosswalk describes rank correlations, not mechanism. Within these limits, temperature dominated the retained associations, and PF01638 showed the same direction in the AEF crosswalk; the data do not establish adaptation or selection.

## Resource availability

### Lead contact

Further information and requests for resources and reagents should be directed to and will be fulfilled by the lead contact, Kourosh Salehi-Ashtiani (ksa3@nyu.edu).

### Materials availability

This study did not generate new unique reagents or materials.

### Data and code availability

• Data: Raw sequencing reads are available through NCBI: BioProject PRJNA929663. The original and decontaminated UAE assemblies are available at Zenodo: 10.5281/zenodo.7758507. Source data and analysis summaries are provided in Tables S1, S2, and S3; Data S1 and S2; Data S1, S2, and S3 ( Zenodo: 10.5281/zenodo.17775003). Figures S1 and S2 and their legends are provided in the consolidated supplemental information. Data reported in this paper will be shared by the lead contact upon request.
• Code: This paper reports original analysis code. Scripts for the primary Google Earth Engine and AlphaEarth Foundations workflows, figure generation, workflow documentation, and an SHA-256 code inventory are publicly available^63^ in GitHub release v1.0.2 at GitHub: https://github.com/olympus-terminal/macroalgae-genome-environment-code/releases/tag/v1.0.2. Scripts, authenticated inputs and outputs, software requirements, and provenance manifests for the post hoc HYCOM sensitivity analysis are supplied as Data S3.
• All other items: Any additional information required to reanalyze the data reported in this paper are available from the lead contact upon request.

## Acknowledgments

We would like to thank the NYUAD sequencing core of the Core Technologies Platform (CTP) and the NYUAD Bioinformatics core. Computational analyses were performed in part using the Jubail high-performance computing cluster at New York University Abu Dhabi and computational resources provided by the A2S2 laboratory. This research was supported by NYUAD Faculty Research Funds (AD060) and Tamkeen under the NYU Abu Dhabi Research Institute Award to the NYUAD Center for Genomics and Systems Biology (73 71210 CGSB9).

## Author contributions

K.S.-A. and A.M. conceptualized the study. A.M., N.A.-M., and J.A.B. collected the field macroalgal samples. L.N. took and edited the herbarium photos. C.R.-M., M.S., and D.H.G. extracted DNA. K.S.-A., A.M., D.C.E.-A., A.K.J., and D.R.N. designed computational data analysis. A.M., K.S.-A., D.C.E.-A., A.K.J., D.R.N., and N.D. performed computational data analysis. A.M., D.R.N., S.T.E., and M.A.-H. did statistical data analysis. A.M., D.C.E.-A., and D.R.N. prepared the figures. A.M., K.S.-A., D.R.N., and D.C.E.-A. wrote the manuscript with input from all authors. K.S.-A. supervised the study.

## Declaration of interests

The authors declare no competing interests.

## Declaration of generative AI and AI-assisted technologies in the manuscript preparation process

The authors used OpenAI Codex,^64^ a generative artificial intelligence (AI) tool, to assist with code development, consistency checks, and language editing. The exact Codex model/version was not retained in the archived project records. All analyses were conducted on the study data. The authors reviewed and verified the code, numerical results, references, and text and take responsibility for the manuscript.

## STAR★Methods

### Key resources table

**Table:** 

REAGENT or RESOURCE	SOURCE	IDENTIFIER
**Biological samples**		

Nine UAE macroalgal genomes	This paper	https://www.ncbi.nlm.nih.gov/bioproject/PRJNA929663; https://doi.org/10.5281/zenodo.7758507

**Deposited data**		

Raw sequencing reads	This paper	https://www.ncbi.nlm.nih.gov/bioproject/PRJNA929663
Original and decontaminated UAE assemblies	This paper	https://doi.org/10.5281/zenodo.7758507
Analysis source data and summaries	This paper	Table S1. Genome inventory, assembly metadata, and analysis sets, Table S2. Primary named-variable Google Earth Engine (GEE) discovery and sensitivity analyses, with HYCOM surface-temperature product sensitivity, Table S3. Secondary AlphaEarth Foundations (AEF) association and site-alignment analyses; Data S1 and S2 (Zenodo concept https://doi.org/10.5281/zenodo.17775003) Data S3 supplied with this revision
Public analysis code	Nelson et al.^63^	https://github.com/olympus-terminal/macroalgae-genome-environment-code/releases/tag/v1.0.2

**Software and algorithms**		

CLC Genomics Workbench	QIAGEN^65^	Version not retained in archived records
ABySS	Simpson et al.^66^	Version not retained in archived records
SPAdes	Bankevich et al.^67^	Version not retained in archived records
BLEACH	Nelson et al.^32^	Pipeline described in cited source
DIAMOND	Buchfink et al.^68^	Version not retained in archived records
FastTree	Price et al.^38^	2.1.11
Python	Python Software Foundation	3.9.23 (Figure 2 rendering)
Biopython	Biopython Project	1.85 (Figure 2 rendering)
Matplotlib	Matplotlib Development Team	3.9.4 (Figure 2 rendering)
HMMER	hmmer.org^69^	3.3.2 (121 genomes); 3.3 (five genomes)
PyHMMER	Larralde and Zeller^70^	0.11.0
SciPy	Virtanen et al.^56^	1.11.4
statsmodels	Seabold and Perktold^71^	0.14.5

**Other**		

MODIS Aqua Level-3 Standard Mapped Image	NASA Ocean Biology DAAC^44^	NASA/OCEANDATA/MODIS-Aqua/L3SMI
ETOPO1	NOAA National Centers for Environmental Information^72^	https://doi.org/10.7289/V5C8276M
AlphaEarth Foundations Satellite Embedding	Google and Google DeepMind^34^^,^^35^	GOOGLE/SATELLITE_EMBEDDING/V1/ANNUAL; archived model/dataset version not retained
HYCOM sea temperature and salinity	National Ocean Partnership Program/Google Earth Engine^42^^,^^43^	HYCOM/sea_temp_salinity; experiment 930
Pfam-A	InterPro/Pfam^36^^,^^41^	Pfam-A36

### Experimental model and study participant details

#### Study design and analysis units

The 13-variable exact-ID GEE screen is the primary named-environment analysis. All 84 global-BH GEE pairs entered a selected-family sensitivity analysis using unique-site means or exact-coordinate-clustered covariance. The 64-component AEF screen is secondary; four priority Pfam–AEF pairs plus PF00092 across all axes formed its separate 68-pair sensitivity family. A unique-site 64 × 13 AEF–GEE analysis provided the site-level cross-representation crosswalk. Figure 4 separately summarizes within-phylum Pfam associations with recorded temperature, latitude, and longitude.

#### Sample collection and species identification

To supplement the global dataset with genomes from a hypersaline subtropical setting, nine UAE genomes were sampled during winter months at three recorded coordinate pairs used for environmental-data extraction. Specimen records disagree on some locality labels (including Ras Ghurab versus Dhabiya), depth fields, and the identity or locality represented by panel I; these fields are reported as unresolved. Table S1A reports the collection records, and Table S1F identifies the nine genomes in the 126-genome analysis set.

In the exact-ID analysis metadata, the nine UAE genomes are labeled six Ochrophyta, two Rhodophyta, and one Chlorophyta. Figure 1 separately retains the unresolved specimen identity/locality in panel I and two raw-versus-published taxon conflicts; the photograph record was not used to reconstruct an analysis phylum label. Macroalgae from these genera and regions have previously been documented in UAE and Iranian waters.^73^^,^^74^ Notably, *Padina* and *Sargassum* specimens were sampled from degraded substrates on recently bleached coral reefs, and *Avrainvillea amadelpha* has been reported as a newly introduced potential invader in the Mediterranean.^40^ These genomes were treated as public data in prior comparative analyses; however, this study provides their primary description.^32^

### Method details

#### Deoxyribonucleic acid (DNA) extraction and sequencing

To mitigate contamination from natural symbioses (bacteria, fungi, endophytes) and extraction inhibitors (polysaccharides, polyphenols), we selected the least-affected tissues and treated samples with 0.1% sodium dodecyl sulfate (SDS) prior to lysis. Genomic DNA was extracted using a modified cetyltrimethylammonium bromide (CTAB)-based phenol–chloroform protocol. Whole-genome sequencing libraries were prepared and sequenced at New York University Abu Dhabi (NYUAD) on an Illumina NovaSeq platform with 150-base-pair (bp) paired-end reads, targeting approximately 100× coverage per genome (National Center for Biotechnology Information (NCBI) BioProject: PRJNA929663; Table S1).

#### Assembler benchmarking and genome assembly

To select an assembly strategy, we benchmarked CLC Genomics Workbench, Assembly By Short Sequences (ABySS), and St. Petersburg genome assembler (SPAdes) using raw reads from eight macroalgae and the microalga *Chlamydomonas reinhardtii* (Table S1B).^65^^,^^66^^,^^67^ ABySS and SPAdes were executed on high-performance computing systems, while CLC was run locally. We compared assembly N50 values—the contig length at which half of the assembly length is contained in contigs of that length or longer—across recorded k-mers 40–60. CLC had the highest recorded N50 in six of nine benchmark records, ABySS in three of nine, and SPAdes in none; SPAdes output was unavailable for two records. Table S1B retains every recorded N50 and missing value. A complete machine-readable per-assembler BUSCO benchmarking matrix was not retained, so the archived BUSCO assembler plot and unsupported quantitative comparison were removed rather than reconstructed.

Based on the traceable N50 benchmark, the final nine UAE genomes were assembled using CLC Genomics Workbench.^65^ The CLC version was not retained in the archived project records. Reads were trimmed to remove adapters, terminal bases, homopolymers, and polynucleotide runs. Assemblies used a k-mer sweep (40, 50, 60), a minimum contig length of 500 bp, and paired-distance autodetection. To remove non-target-organism contigs, all assemblies underwent six iterative rounds of the BLEACH (Basic Local Alignment Search Tool [BLAST]-Limiting Eukaryotic Assembly Contamination, Heuristically) pipeline.^32^ The pipeline used DIAMOND protein-alignment mode (protein BLAST, BLASTP)^68^ against the NCBI non-redundant protein sequence database (nr).^75^

#### Dataset compilation and quality assessment

The nine newly described UAE genomes were integrated with 117 publicly available macroalgal genomes. The mean Benchmarking Universal Single-Copy Orthologs (BUSCO) completeness score for the 126-genome cohort was 68.8 ± 14.9% present (Table S1F). BUSCO completeness used the Eukaryota dataset with AUGUSTUS.^62^^,^^76^ BUSCO and related universal-ortholog assessments provide informative but model-dependent summaries of assembly and gene-repertoire completeness.^77^^,^^78^ Complementary repertoire-aware tools characterize completeness as a gene-set quality assessment.^79^ Raw Pfam counts were the primary measure; targeted sensitivity analyses modeled BUSCO as a continuous covariate and used explicit ≥50% and ≥70% dataset-composition thresholds.

Examples of reference genomes in this study included *Ectocarpus siliculosus* (Phaeophyte, approximately 196 megabase pairs (Mbp));^10^ *Ectocarpus subulatus* (Phaeophyte, approximately 242 Mbp);^11^ *Saccharina japonica* (Phaeophyte, approximately 535 Mbp);^80^ *Chondrus crispus* (Rhodophyte, approximately 104.98 Mbp);^12^ *Gracilariopsis chorda* (Rhodophyte, approximately 92.18 Mbp);^13^ *Porphyra umbilicalis* (Rhodophyte, approximately 87.89 Mbp);^14^ *Neopyropia yezoensis* (Rhodophyte, approximately 107.59 Mbp);^15^ *Neoporphyra haitanensis* (Rhodophyte, approximately 53.25 Mbp) and *Gracilariopsis lemaneiformis* (Rhodophyte, approximately 88.69 Mbp);^16^ *Kappaphycus alvarezii* (Rhodophyte, approximately 336.72 Mbp);^17^ *Caulerpa lentillifera* (Chlorophyta, approximately 28.77 Mbp);^18^ *Ulva mutabilis* (Chlorophyte, approximately 98.48 Mbp)^19^; and *Chara braunii*.^20^ Table S1D provides the 14-genome reference inventory and accession reconciliation.

#### Phylogenetic analysis

Phylum-specific queries for the ribulose-1,5-bisphosphate carboxylase/oxygenase large-subunit (RbcL) protein, encoded by *rbcL*, were used for marker retrieval. The deposited tree contains 119 tips and was inferred with FastTree 2.1.11^38^ under the Jones–Taylor–Thornton amino acid substitution model with a 20-category approximation of among-site rate variation (JTT+CAT20); node labels report Shimodaira–Hasegawa-like local support. Accession-gated reconciliation yielded 116 safe mappings, and 112 tips occurred in the 126-genome analysis set used for topology-aware sensitivity. For Figure 2, deposited tips were reconciled by exact tree-label, query-sequence, accession, and metadata checks against Table S1 and the retained integrity audit. The display was restricted to 96 tips with a unique accession-gated authoritative record, complete displayed metadata, a species-level binomial, and no unresolved cross-source taxonomy or identity conflict. The 23 omitted tips comprised 12 genus-only names, three without a safe metadata mapping, and eight with unresolved conflicts. Display-only pruning was followed by ladderization without rerooting; the induced topology and support values were inherited from the full 119-tip inference, and pairwise patristic distances among displayed tips were preserved. The figure was rendered in Python 3.9.23 using Biopython 1.85 and Matplotlib 3.9.4, with character codes duplicating color encodings for grayscale accessibility.

Analyses are shown for pooled genomes and as phylum-stratified discovery summaries. Phylum labels provide a coarse grouping, and the topology-eigenvector sensitivity models accession-gated tree structure.

#### Protein family annotation and analysis

HMMER hmmsearch outputs from Nelson et al.^32^ supplied the archived Pfam-search records. Headers document HMMER 3.3.2 for 121 genomes and HMMER 3.3 for five genomes,^69^ using Pfam-A^36^ and a sequence E-value threshold of 1 × 10^−5^. Genome-level reconstruction yielded 10,707 strict, variable Pfam accessions across 126 genomes. Pooled correlations tested all 685,248 Pfam–AEF pairs. The three phyla were represented unequally (Table S1). Figure 4 reports the phylum-stratified recorded-temperature, latitude, and longitude analyses with sample-size–weighted Stouffer meta-analysis^47^ and I^2^ heterogeneity summaries.^48^ Table S3B reports the phylum-centered AEF screen, and Table S3F reports the within-phylum AEF screens. These summaries address broad phylum imbalance, while targeted sensitivities model topology, spatial clustering, and genome quality.

Pfam abundances for the discovery and targeted analyses were reconstructed directly from per-genome hmmsearch outputs, producing 2,072,937 sequence-level Pfam-hit records across 126 genomes and 10,707 strict, variable accessions. The pooled, phylum-centered, and within-phylum AEF screens use these reconstructed raw counts.

Table S1F defines the 126-genome analysis set, and Table S1G identifies the reconstructed raw Pfam matrix. Table S2 reports the exact-ID GEE discovery screen and its 84-pair selected-family sensitivity analysis. Table S3 reports the secondary pooled, phylum-centered, within-phylum, selected-family, and site-level AEF–GEE alignment results together with output hashes.

#### Recorded metadata–Pfam discovery analysis

Figure 3 tested raw counts for 10,707 strict Pfam accessions against World Ocean Atlas 2018 (WOA2018) recorded temperature,^46^ latitude, longitude, and the prespecified ordinal environment category Freshwater = 0, Brackish = 1, and Marine = 2 across the same 126 genomes. Two-sided Spearman tests formed one 42,828-pair family for global Benjamini–Hochberg and Bonferroni correction. Panel B displays the ten smallest raw *p* values for the recorded environment category as log2(mean raw count +1), and panel C displays raw log2(count +1) profiles for all 41 category-associated Pfams meeting global q < 0.05. Average-linkage Euclidean clustering was applied to row-z-scored log2(count +1) profiles with deterministic optimal ordering.

#### Google Earth Engine extraction and exact-ID matching

Google Earth Engine (GEE) is a cloud-based platform for server-side geospatial analysis.^33^ The GEE analysis used Moderate Resolution Imaging Spectroradiometer (MODIS) Aqua ocean products from Earth Engine collection NASA/OCEANDATA/MODIS-Aqua/L3SMI^44^ and ETOPO1-derived depth and distance to coast.^72^ The products have different native nominal resolutions, but the script combined 13 bands and requested reducer.first evaluation at scale = 1,000 m. The requested evaluation scale did not increase the native information content of the nominal 4-km MODIS or 1-arc-minute ETOPO1 sources, and the archived script did not explicitly set a resampling kernel. The dynamic collections were filtered from 2020 to 01-01 through 2023–12-31 end-exclusive. The complete 126-ID extraction reports the observed coverage pattern, and the 2020–2023 summaries provide coordinate-linked environmental context.

Sea surface temperature (SST) variables were derived from National Aeronautics and Space Administration (NASA) MODIS Aqua Level-3 Standard Mapped Images at nominal 4-km resolution.^45^ The analysis filters from 2020 to 01-01 through 2023–12-31 end-exclusive and computes temporal mean, minimum, and maximum, derives maximum minus minimum as range, and computes June–August and December–February means. Annual SST fields were available for 107 genomes, summer SST for 83, and winter SST for 95; Table S2 uses these exact-ID rows as an exploratory discovery screen. These summaries describe coordinate-linked conditions during the stated 2020–2023 interval.

Ocean-color variables used MODIS Aqua chlorophyll-a products with the Ocean Color 3-band algorithm for MODIS (OC3M)^81^ and particulate organic carbon products based on published ocean-color relationships.^82^ The exact-ID extraction provided ocean-color values for 72 genomes while retaining source-product missingness.

Bathymetry was derived from the bedrock band of Earth Engine image NOAA/NGDC/ETOPO1 as a static bathymetry layer at 1-arc-minute nominal resolution.^72^ Bathymetry differs in scale and temporal meaning from the dynamic MODIS products. The exact-ID extraction returned depth and distance to coast for all 126 genomes. Depth and distance to coast entered the exploratory screen as recorded geospatial descriptors.

Reconstructed raw HMM counts were filtered before environmental testing to 6,703 Pfams present in at least 7 of 126 genomes (5%). Spearman correlations required at least 10 valid pairs. The 6,703 Pfams crossed with 13 variables define 87,139 possible combinations. Sixteen were nonestimable because the Pfam vector was constant within the variable-specific complete-case subset: PF07072 for summer SST, and PF08734, PF13464, and PF19489 for each of chlorophyll mean, chlorophyll maximum, chlorophyll standard deviation, particulate organic carbon mean, and water clarity. The remaining 87,123 estimable tests (6,700–6,703 per variable) entered global Benjamini–Hochberg and Bonferroni correction. Tables S2A–S2C report the exact-ID inputs, variable-level accounting, and results.

#### Phylum-stratified discovery meta-analysis

For the 126-genome Figure 4 analysis, we computed Spearman coefficients within each phylum contributing an estimable pair. Each two-sided *p* value was converted to a signed *Z* score, sign(ρ) × Φ^−1^(1 − p/2), and combined by weighted Stouffer analysis with weights √n.^47^ In parallel, within-phylum Spearman coefficients were Fisher-transformed and pooled with inverse-variance weights (*n* − 3) to calculate Cochran’s Q^48^ and I^2^. Benjamini–Hochberg correction was applied across the 16,797 eligible meta-analysis tests, each represented by at least two phyla.

For the 126-genome Figure 4 analysis, heterogeneity was calculated as I^2^ = max[0, (Q − df)/Q] × 100%, where Q is Cochran’s Q and df denotes degrees of freedom.^83^ We classified a pair as nominal shared-direction, low-heterogeneity when its Stouffer p was <0.05, all contributing nonzero within-phylum coefficients shared the same direction, and I^2^ was <50%. The FDR-supported shared-direction category additionally required Benjamini–Hochberg q < 0.05.

#### Data extraction pipeline: Technical implementation

In the exact-ID extraction, the 126 recorded coordinate records were converted to point features while preserving canonical genome IDs. The extraction applied the original interval from 2020 to 01-01 through 2023–12-31 end-exclusive, target bands, temporal reducers, and combined stack. Returned IDs and coordinates were checked against the input at 1 × 10^−10^ precision; this was a transfer-integrity check, not an estimate of spatial accuracy. Canonical IDs were joined one-to-one, sampling used the recorded coordinates, and source-product missing values remained missing.

#### Quality control for GEE analyses

Source-product no-data pixels were retained as missing, and sampling used the recorded coordinates. SST annual statistics were available for 107 genomes, summer SST for 83, winter SST for 95, ocean-color variables for 72, and depth and distance to coast for all 126. Each correlation used all available matched observations at the recorded coordinates.

#### Derived environmental variables

The extraction contains the same 13 intended bands: SST mean, minimum, maximum, range, summer mean, and winter mean; chlorophyll-a mean, maximum, and standard deviation; particulate organic carbon mean; ETOPO1 depth; ETOPO1-derived distance to coast; and the mean MODIS ratio of remote-sensing reflectance at 443 to 555 nm [Rrs(443)/Rrs(555)] used as a water-clarity ratio. The summaries from 2020 to 01-01 through 2023–12-31 end-exclusive provide coordinate-linked environmental context.

#### Primary selected-family sensitivity analysis for exact-ID GEE associations

The discovery screen used one-to-one canonical genome identifiers, the 13 named variables, reconstructed raw HMM counts, and the prespecified 5% Pfam-prevalence rule. Global Benjamini–Hochberg and Bonferroni correction covered all 87,123 estimable tests (Tables S2B–S2C). The selected post hoc family was then fixed as all 84 pairs meeting discovery-wide global q < 0.05; effect sizes, confidence intervals, adjusted values, and the structured null are reported in Tables S2E–S2F.

For each selected GEE pair, seven required specifications tested consistent direction and selected-family q < 0.05: raw-count unique-site Spearman, total-hit-normalized unique-site Spearman, peptide-normalized unique-site Spearman, the total-hit-normalized quality/phylum model with coordinate-clustered covariance, the total-hit-normalized BUSCO ≥50% unique-site analysis, the total-hit-normalized three-eigenvector topology model with coordinate-clustered covariance, and the total-hit-normalized structured null. The quality/phylum model adjusted for log10 assembly nucleotide count, BUSCO completeness, log10 total Pfam hits, log10 final peptide-record count, and phylum; the topology model used the same continuous quality covariates plus three unit-edge phylogenetic eigenvectors selected by an outcome-blind broken-stick rule^84^ and omitted phylum terms. Both models clustered covariance by exact coordinate. All coordinate-clustered regressions and Benjamini–Hochberg calculations used statsmodels 0.14.5.^71^ Site bootstrap intervals used 999 resamples. BUSCO ≥70% and five- and ten-eigenvector topology fits supplied additional sensitivity estimates.

The GEE structured null used 99,999 permutations of intact coordinate-site labels within site phylum-composition strata. Lower and upper randomization-tail probabilities each used the plus-one convention^85^; the two-sided *p* value was twice the smaller tail, capped at 1. Benjamini–Hochberg correction was applied across all 84 selected pairs separately for each specification. Figure 5 displays all 84 pairs and the seven required results.

#### Post hoc HYCOM surface-temperature product sensitivity

We queried the National Ocean Partnership Program (NOPP) data-assimilative Hybrid Coordinate Ocean Model collection distributed through Earth Engine as HYCOM/sea_temp_salinity (experiment 930; catalog accessed August 4, 2026)^42^^,^^43^ from 2020 to 01-01 inclusive through 2023–12-31 exclusive. The filtered collection contained 11,677 images. We selected the 0-m water_temp_0 band, converted source values to degrees Celsius as raw × 0.001 + 20, and calculated the temporal mean, minimum, maximum, full-window maximum-minus-minimum range, June–August mean, and December–February mean. We sampled each summary with ee.Reducer.first at the recorded point on the stored native EPSG:4326 band grid (catalog pixel size, 8,905.6 m), without requesting a finer grid, buffering coordinates, relocating points to another ocean cell, or imputing source-product no-data. All six summaries were available for 52 of 126 genomes at 31 of 90 exact sites (Table S2I).

For exact-site product agreement, we compared each HYCOM summary with its MODIS counterpart by two-sided Spearman correlation across pairwise-complete exact sites and applied Benjamini–Hochberg correction across the six comparisons. For the Pfam product sensitivity, we fixed the family as the 73 SST–Pfam pairs already selected by the primary GEE screen. At each coordinate, the outcome was the arithmetic mean observed raw Pfam count among genomes sharing that site; tests used sites containing both mapped products and required at least 10 sites. We applied Benjamini–Hochberg correction once across all 73 HYCOM *p* values, representing an unestimable test by *p* = 1. A pair met the product rule when HYCOM q < 0.05 and its coefficient matched the original MODIS raw-count site-effect direction. The fixed 43-pair retained and 13-pair global-Bonferroni subsets used these 73-test-adjusted q values and were not readjusted separately (Tables S2J–S2K).

For the native-cell sensitivity, we assigned exact coordinates to stored HYCOM cells using the source affine grid transform. We first averaged genomes within each exact site and then gave exact-site means equal weight within each native cell: Pfam cell outcomes were means of exact-site raw-count means, MODIS cell values were means of exact-site MODIS values, and the single stored HYCOM cell value was retained. Product correlations used 25 cells for five summaries and 21 cells for summer. Two-sided Spearman tests required at least 10 cells; Benjamini–Hochberg correction was applied separately across the six product comparisons and across all 73 native-cell Pfam tests, with unestimable tests represented by *p* = 1. The same direction-and-q rule and fixed subsets were used (Tables S2L–S2M).

#### Secondary AEF representation

We analyzed the precomputed 64-dimensional Google Earth Engine collection GOOGLE/SATELLITE_EMBEDDING/V1/ANNUAL (Satellite Embedding V1).^35^ The primary AlphaEarth Foundations paper^34^ describes the Space Time Precision encoder and reports evaluation results for AEF model v2.0. Its documented inference inputs are time-stamped Sentinel-2 Level-1C optical,^86^ Sentinel-1 ground-range-detected radar, and Landsat 8/9 optical and thermal imagery; additional radar, elevation, light detection and ranging, climate, gravity, land-cover, and geocoded-text sources serve as training targets. The training loss combines source-specific reconstruction, batch-uniformity, missing-input-consistency, and text–video contrastive terms. We sampled the precomputed Earth Engine product rather than running the model.

Sentinel-2 mission context is described in the source documentation.^86^ The AEF catalog identifies the product as a multi-source 64-dimensional annual embedding field with nominal 10-m pixels (GOOGLE/SATELLITE_EMBEDDING/V1/ANNUAL). The catalog reports coverage of shallow, intertidal, reef, and coastal-water settings, but the published evaluations do not validate submerged macroalgal habitats. Land adjacency, water-column effects, tide, mixed pixels, and coordinate error can affect the embedding values at coastal sites. We therefore interpret the embeddings only as coordinate-linked latent geospatial descriptors.

The archived AEF extractor mosaicked the unfiltered annual ImageCollection without selecting a year; overlap priority followed collection order. It then applied reduceRegions with a mean reducer at nominal 10-m scale to the complete sampling-location FeatureCollection. The retained values define the coordinate mosaic used in the secondary analyses. The archived table stores genome identifiers, coordinates, and A00–A63 but not the collection year, model version, processing-software version, or dataset version; an exact internal layer version therefore cannot be assigned retrospectively.

#### Site-level alignment of AEF with named GEE variables

The exact-ID AEF and GEE tables were joined across all 126 genomes and collapsed to 90 unique coordinate sites after verifying that, within each repeated coordinate, genome records had identical AEF values and identical GEE values. Pairwise-complete two-sided Spearman correlations were calculated for all 64 AEF components against all 13 named GEE variables (832 pairs; 48–90 sites per pair). Benjamini–Hochberg and Bonferroni correction covered the complete 832-pair family.

The 64 AEF components (A00–A63) were treated as a jointly learned representation. The site-level AEF–GEE correlations provide a descriptive cross-representation crosswalk to named variables; they do not decode individual axes or provide independent validation. Axis meanings remain distributed and nonunique, and coefficients and global multiplicity-adjusted values describe only the observed alignment.

#### Phylum–environment biogeography

The three phyla are unevenly distributed across the recorded geographic and temperature metadata, creating strong lineage–environment covariance. Within-phylum centering estimates covariance around each phylum mean, and the topology-eigenvector analysis provides the accession-gated relatedness sensitivity.

Within-phylum residualization subtracts the phylum mean from each Pfam profile and estimates covariance between deviations from that broad group mean and an AEF axis. The topology-based eigenvector sensitivity addresses finer tree structure for accession-gated matches.

#### Secondary Pfam–AEF correlations

For each of 10,707 strict Pfam accessions, reconstructed raw counts across 126 genomes formed the discovery abundance profile. Each genome was paired by exact genome ID with the 64 AEF values in the coordinate-linked analysis table.

Pairwise Spearman rank correlations were computed between each Pfam abundance vector and each AEF dimension, producing a genome–environment association matrix. Spearman’s ρ was used because Pfam copy numbers are non-normally distributed, include zeros, and may relate monotonically but nonlinearly to the embeddings. Each test yielded ρ and a two-sided *p* value. Benjamini–Hochberg correction across the complete Pfam–AEF discovery matrix produced global q values, with global q < 0.05 used as the discovery threshold. We treated the matrix as an exploratory description of covariance rather than a physical interpretation of individual axes.

#### Hierarchical clustering of Pfam and AEF correlation profiles

We calculated Spearman rank correlations between reconstructed raw Pfam counts and 64 AEF axes across 126 exact-ID genomes. Each 64-axis correlation profile was *Z* score normalized across axes for display and clustering; inferential outputs retain raw Spearman coefficients. SciPy 1.11.4^56^ average-linkage hierarchical clustering with correlation distance organized all 10,707 profiles and all 64 axes. The duplicate-profile audit identified 8,713 unique profiles and 1,994 additional accessions sharing an exact profile; every accession remained in Figure S2. Discovery-screen *p* values were adjusted with the Benjamini–Hochberg procedure.^37^ The selected post hoc q values were computed with the same procedure^37^ over the explicitly defined 68-pair sensitivity family.

#### Secondary AEF selected-family sensitivity analyses

The AEF selected family contained 68 pairs: PF13411–A18, PF01638–A52, PF01638–A53, PF10988–A36, and PF00092 against all 64 axes. The three abundance specifications were raw sequence-level HMM hit counts, counts per total Pfam hit, and counts per final peptide record. Peptide denominators were available for 121 genomes. The ratio specifications estimate relative genome composition, while raw counts remain the primary copy-number measure.

PF00092 sequence classification used exact peptide sequences recovered for 2,024 of 2,128 PF00092.31 protein-hit records. We searched these proteins with PyHMMER 0.11.0^70^ using the pinned PF00092.35 HMM at its model-specific gathering threshold and classified residues aligned to states 7, 9, 11, 79, and 112. D/S/S/T/D defined a complete canonical signature. Control sequences for PDB: 1SHU^52^ and PDB: 1AO3^51^ were obtained from the Research Collaboratory for Structural Bioinformatics Protein DataBank (RCSB PDB)^87^; the aligned control signatures were DSSTD and DSSST, respectively.

For the AEF selected family, rank effects were calculated from unique-site means or genome-level rank models with covariance clustered by exact coordinate. Continuous assembly size, BUSCO completeness, total Pfam hits, peptide-search-space size, and phylum entered the quality models. BUSCO ≥50% and ≥70% subsets provided dataset-composition sensitivities, and site bootstrap intervals used 999 unique-site resamples.

The AEF topology sensitivity used the accession-gated 112-genome subset at 82 sites. Unit-edge topological distances were converted to phylogenetic eigenvectors; an outcome-blind broken-stick rule^84^ selected three axes, with five- and ten-axis fits as additional sensitivity estimates.

The AEF structured null used 99,999 permutations of intact coordinate-site labels within site phylum-composition strata. Lower and upper plus-one randomization tails^85^ were calculated, and the two-sided *p* value was twice the smaller tail, capped at 1. Benjamini–Hochberg correction was applied across the 68 selected pairs separately for each abundance representation. A pair passed the seven required specifications when every effect direction was consistent and selected-family q < 0.05 for raw-site, total-hit-site, peptide-site, total-hit quality, total-hit BUSCO ≥50%, total-hit three-eigenvector topology, and total-hit structured-null results.

#### Within-phylum AEF–Pfam screens

Within-phylum Pfam–AEF screens used analytic two-sided Spearman *p* values followed by a global Benjamini–Hochberg correction for each stated screen. Chlorophyta, Ochrophyta, and Rhodophyta screens required at least three nonzero observations per Pfam; a separate Rhodophyta sensitivity required at least ten nonzero observations. Every eligible Pfam was tested against all 64 AEF axes.

The minimum-three-nonzero screens evaluated 304,704 Chlorophyta, 374,784 Ochrophyta, and 459,072 Rhodophyta pairs; their minimum global q values were 0.3148, 0.0592, and 0.0916, respectively. The Rhodophyta minimum-ten-nonzero sensitivity evaluated 328,320 pairs and had minimum global q = 0.0655. Each screen yielded zero pairs at global q < 0.05 (Table S3F).

### Quantification and statistical analysis

Unless otherwise specified, association tests were two-sided and used Spearman rank correlation with pairwise-complete observations. Source-product no-data values remained missing and were not imputed. The recorded-metadata discovery family comprised 42,828 protein family (Pfam)–variable tests. For the primary exact genome identifier Google Earth Engine (GEE) screen, Pfams present in at least seven of 126 genomes were tested against 13 variables. Of 87,139 possible combinations, 16 were nonestimable because the Pfam vector was constant in the variable-specific complete-case subset; the remaining 87,123 tests entered global Benjamini–Hochberg and Bonferroni correction.

Post hoc selected-family sensitivity analyses were restricted to the 84 GEE pairs meeting discovery-wide global q < 0.05 and, separately, the 68 selected AlphaEarth Foundations (AEF) pairs defined above. Repeated coordinates were addressed using unique-site means or covariance clustered by exact coordinate. The required specifications evaluated abundance representation, assembly quality, phylogenetic topology, and a structured null. Structured-null *p* values used 99,999 permutations of intact coordinate-site labels within site phylum-composition strata, plus-one randomization tails, and separate Benjamini–Hochberg correction within each stated selected family. A pair was retained only when its direction matched discovery and selected-family q < 0.05 in all seven required specifications.

Phylum-stratified recorded-metadata effects were combined using weighted signed Stouffer analysis, with heterogeneity summarized by I-squared. Within-phylum AEF screens, the 832-pair site-level AEF–GEE crosswalk, and the six HYCOM–MODIS product comparisons each used the multiplicity family stated in the corresponding Methods subsection. Rank statistics were calculated with SciPy 1.11.4, and coordinate-clustered regression models were fitted with statsmodels 0.14.5. Exact inputs, sample sizes, effect estimates, *p* values, adjusted q values, and sensitivity outputs are reported in Tables S1, S2, and S3 and Data S3.

## Supplemental information

Document S1. Figures S1 and S2

Table S1. Genome inventory, assembly metadata, and analysis sets The workbook defines canonical genome identifiers and membership in each analysis set. Missing values remain missing. Table S1A. United Arab Emirates (UAE) genome metadata. Collection and assembly metadata for nine UAE genomes. Table S1B. Assembly N50 benchmarking. N50 values (bp) are reported for 9 records: eight macroalgae and one microalga, Chlamydomonas reinhardtii. CLC Genomics Workbench and Assembly By Short Sequences (ABySS) were evaluated at k-mer sizes 40, 50, and 60; the recorded St. Petersburg genome assembler (SPAdes) N50 is shown where available. For each record, the highest CLC and ABySS N50 across the three k-mer settings was compared with the recorded SPAdes N50. CLC had the highest recorded N50 for 6/9 genomes, ABySS for 3/9, and SPAdes for 0/9; SPAdes N50 was unavailable for 2/9. Table S1A contains retained-assembly Benchmarking Universal Single-Copy Ortholog (BUSCO) values, but no complete machine-readable per-assembler BUSCO benchmarking matrix was present in the audited workbook sources; per-assembler BUSCO results are therefore not reported or reconstructed from historical figure PDFs. Table S1C. RbcL phylogeny inventory and reconciliation. Taxonomy, marker sequence, and identifier mapping are reported for 120 inventory records. Of these, 119 had an RbcL sequence and matched the 119- record alignment and deposited 119-tip tree; ID 47 (RbcL = NA) was the sole inventory record absent from both. Exact accession and species reconciliation yielded 116 safe one-to-one metadata mappings, including 112 genomes in Table S1F and 4 source-only records; IDs 58, 105, and 108 remain unsafe for master-metadata mapping. Genome ID cells for IDs 97–103, 106, and 107 were corrected from same-ID master evidence. The reconciliation audit records all 120 inventory rows and unresolved boundaries; the taxonomy audit records exact accepted World Register of Marine Species (WoRMS) results, AphiaIDs, source URLs, and access times for six recorded names without inferring sequence or organism identity. Table S1D. Reference genomes. Published reference-genome metadata for 14 genomes. Table S1E. Candidate-genome source inventory (131 records; not an analysis cohort). Table S1F defines the 126-record accession-gated cohort used in all manuscript analyses, with analysis-specific missingness reported separately. The five Table S1E-only records lack retained AlphaEarth Foundations embeddings and are flagged aef_present = FALSE, aef_coordinate_status = NOT_IN_AEF, and safe_for_aef_pfam_analysis = FALSE. Table S1G indexes the reconstructed raw protein family (Pfam) count matrix. Table S1F. Macroalgal genome analysis set. The 126-genome dataset includes canonical genome identifiers, collection coordinates, metadata, matched Pfam profiles, and 64 AEF components. The AEF components form a collective, dimensionless embedding vector and were analyzed as unitless latent axes. Table S1G. Raw Pfam input index. Path and hash for the 126-genome matrix reconstructed from per-genome profile hidden Markov model searches performed with HMMER hmmsearch. Raw domain-hit counts are the primary abundance measure. The Figure 3 recorded-metadata screen comprised 42,828 tests; 491 had p < 0.001, 228 had global Benjamini-Hochberg (BH) q < 0.05, and 13 met Bonferroni p < 0.05

Table S2. Primary named-variable Google Earth Engine (GEE) discovery and sensitivity analyses, with HYCOM surface-temperature product sensitivity Exact-genome-identifier (ID) matching joined environmental values and protein family (Pfam) counts for 126 genomes. The GEE discovery family comprised 87,123 tests, with 6,700–6,703 estimable Pfams per named variable; 84 associations representing 51 Pfams met global Benjamini-Hochberg (BH) q < 0.05, and 13 met Bonferroni p < 0.05. The primary retained GEE result set comprises 49 Pfam-environment pairs across 30 Pfam accessions that retained direction and selected-family support in all seven required specifications; all 13 discovery-Bonferroni associations are retained. Tables S2I–K report the exact-site HYCOM product-sensitivity specification, whereas Tables S2L–M report a correlated post hoc equal-weight native-cell sensitivity. Table S2A. Exact-ID environmental extraction. GEE values for 126 canonical genome coordinates from 2020 to 01-01 through 2023–12-31 end-exclusive and 13 environmental bands. Unavailable pixels remain missing. The extraction used a 1-km sampling request and preserved negative coastal bathymetry values at the recorded coordinates. Table S2B. Variable-level summary. Non-missing sample sizes and multiplicity results for 13 environmental variables plus the TOTAL row. Table S2C. Global-BH discovery associations. All 84 raw-count Pfam-GEE pairs meeting global BH q < 0.05 in the 87,123-test exact-ID family. These pairs define the selected family evaluated in Table S2E–G. Table S2D. Analysis output index. Paths, byte sizes, row counts, and 256-bit Secure Hash Algorithm (SHA-256) hashes for the exact-ID GEE extraction, complete discovery family, selected-pair sensitivity analyses, structured null, authenticated InterPro annotations, and the exact-site and equal-weight native-cell HYCOM product-sensitivity specifications. Table S2E. GEE selected-pair sensitivity results. The 2,016 rows comprise 84 discovery pairs across three abundance representations and eight specifications: descriptive genome-level Spearman correlation; unique-site Spearman correlation; a quality/phylum model with site-clustered covariance; BUSCO ≥50% and ≥70% unique-site analyses; and three-, five-, and ten-phylogenetic-eigenvector topology models with site-clustered covariance. BH correction is applied within each specification-by-representation family of 84 pairs. Table S2F. GEE structured-null results. For each of the 84 selected pairs, total-hit-normalized site means were evaluated with 99,999 permutations of intact site environmental labels within site phylum-composition strata. Two-sided *p* values equal twice the smaller conditional randomization tail, including ties, and BH correction spans all 84 selected pairs. Fifty-six associations met corrected q < 0.05. Table S2G. GEE seven-specification candidate summary. All 84 selected discovery pairs, including the seven required specification flags, direction consistency, retained-result status, and authenticated accession-level InterPro fields for the 49 retained pairs. The retained set comprises 49 pairs across 30 Pfam accessions. Table S2H. Retained-Pfam annotation index. One authenticated InterPro record for each of the 30 Pfam accessions represented by the 49-pair primary retained GEE result set. Names and entry types are accession-level database labels. Table S2I. Exact-ID HYCOM surface-temperature extraction. Six summaries—mean, maximum, minimum, full-window maximum-minus-minimum range (the field named hycom_sst_annual_range_c), June-August mean, and December-February mean—were calculated from band water_temp_0 of the National Ocean Partnership Program (NOPP) HYCOM collection distributed through Google Earth Engine as HYCOM/sea_temp_salinity (experiment 930; catalog accessed August 4, 2026: https://developers.google.com/earth-engine/datasets/catalog/HYCOM_sea_temp_salinity; Cummings and Smedstad^42^). Values were sampled at each recorded coordinate from 2020 to 01-01 to 2023–12-31 (end-exclusive), using raw ∗ 0.001 + 20.0 °C. Thus, hycom_sst_annual_range_c is the maximum minus the minimum over the complete 2020–2023 window, not an average annual range. Sampling used the stored HYCOM band grid without coordinate relocation or missing-value imputation. All six summaries were available for 52 of 126 genomes at 31 of 90 exact sites; unavailable cells remain missing. Table S2J. Exact-site MODIS-HYCOM surface-temperature concordance. Each unique recorded coordinate contributed one observation. Spearman correlations compared six matched sea-surface-temperature summaries at 23 sites for summer and 30 sites for each other summary, including the full-window maximum-minus-minimum range. All six correlations were positive and met BH q < 0.05 across the six-test family (rho = 0.901–0.993). Mean and median HYCOM-minus-MODIS differences, mean absolute differences, root-mean-square differences, and product-specific ranges are also reported. Table S2K. Exact-site HYCOM product sensitivity for selected SST-Pfam associations. The 73 GEE-selected SST-Pfam pairs were re-estimated as two-sided Spearman correlations across pairwise-complete exact sites, using the arithmetic mean of observed raw Pfam counts among genomes sharing a coordinate and the matched HYCOM summary. The selected family contains five SST summaries because the full-window range yielded no selected GEE pair. BH correction spans all 73 HYCOM tests; unestimable tests would enter as p = 1, although all were estimable. HYCOM effects matched the authenticated original MODIS site-effect direction for 73 of 73 pairs, and 42 had HYCOM BH q < 0.05. Among the fixed 43-pair seven-check-retained SST subset, 43 directions matched and 30 had q < 0.05; among the fixed 13 discovery-Bonferroni SST pairs, 13 directions matched and 12 had q < 0.05. Neither subset was readjusted separately. HYCOM is a data-assimilative model product sampled at the same recorded coordinates. Table S2L. Equal-weight native-cell MODIS–HYCOM concordance sensitivity. HYCOM-complete coverage comprised 52 genomes, 31 exact sites, and 26 native cells. After exact-site aggregation, each occupied native HYCOM cell received equal weight; the matched comparison used 21 cells for summer and 25 cells for each other summary, with at most 3 exact sites in one cell. All six correlations were positive and met BH q < 0.05 across six tests (rho = 0.930–0.995). Reported differences compare HYCOM with the equal-weight mean of MODIS exact-site values within each native cell. Table S2M. Equal-weight native-cell HYCOM sensitivity for selected SST–Pfam associations. Observed raw Pfam counts were first averaged among genomes at each exact coordinate; the resulting exact-site means were then averaged with equal weight within each occupied native HYCOM cell. All 73 effects retained the authenticated original MODIS site-effect direction, and 42 had native-cell HYCOM BH q < 0.05 across the 73- test family. Among the fixed 43-pair seven-check-retained SST subset, 43 directions matched and 28 had q < 0.05; among the fixed 13 discovery-Bonferroni SST pairs, 13 directions matched and 12 had q < 0.05. Relative to exact-site HYCOM thresholding, 3 pairs gained and 3 lost BH support; 67 were unchanged. Neither fixed subset was readjusted separately

Table S3. Secondary AlphaEarth Foundations (AEF) association and site-alignment analyses The pooled Pfam-AEF screen evaluated 10,707 strict Pfam accessions against 64 unitless latent axes across 126 exact-ID genomes (685,248 tests). The descriptive site-level cross-representation alignment/crosswalk evaluated 64 axes against 13 named GEE variables at up to 90 coordinate sites (832 tests); 224 met global BH q < 0.05 and 44 met Bonferroni p < 0.05. Axis meanings are distributed and nonunique, and Figure S2 displays the complete exploratory Pfam-AEF correlation-profile landscape. Table S3A. Pooled genome-level Pfam-AEF associations. All 24 raw-count Pfam-AEF pairs meeting global BH q < 0.05 in the 685,248-test family, spanning 17 Pfams and 13 AEF axes; three pairs also met Bonferroni p < 0.05. Table S3B. Phylum-centered genome-level Pfam-AEF associations. All 17 pairs meeting global BH q < 0.05 after subtracting each phylum-specific Pfam mean before computing the 126-genome correlations. Table S3C. Site-level AEF associations with recorded metadata. All 192 Spearman correlations of A00-A63 with recorded temperature, latitude, and longitude after giving each of 90 unique coordinate sites one observation. BH and Bonferroni correction span the 192-test family; 51 correlations met BH q < 0.05 and 23 met Bonferroni *p* < 0.05. Table S3D. Site-level recorded-metadata summary by AEF axis. For each unitless axis, the table reports site-level correlations with recorded temperature, latitude, and longitude, the strongest recorded descriptor, and the pooled Pfam-association summary. Table S3E. AEF output index. Paths, byte sizes, row counts, and SHA-256 hashes for the pooled AEF analyses, descriptive site-level cross-representation AEF-GEE alignment/crosswalk, and corrected selected-set sensitivity outputs. Table S3F. Within-phylum specification summary. Numbers of genomes, eligible Pfams, tests, nominal *p* < 0.001 results, global BH q < 0.05 results, and minimum q for the Chlorophyta, Ochrophyta, and Rhodophyta screens and the Rhodophyta ≥10-nonzero sensitivity. The global-BH count was zero in all four specifications. Table S3G. Site-level AEF-GEE alignment. All 832 descriptive cross-representation Spearman correlations between 64 unitless AEF axes and 13 named GEE variables after exact-ID joining and unique-site aggregation. Global BH correction spans all 832 correlations; 224 met q < 0.05 and 44 met Bonferroni *p* < 0.05. This site-level alignment/crosswalk retains distributed, nonunique axis meanings. Table S3H. GEE-variable alignment summary. For each of the 13 named GEE variables, the table reports the number of coordinate sites, the strongest AEF-axis correlation, and counts meeting global BH and Bonferroni thresholds in the 832-test descriptive site-level cross-representation alignment/crosswalk. Table S3I. AEF-axis alignment summary. For each of A00–A63, the table reports its strongest named GEE correlate and counts meeting global BH and Bonferroni thresholds in the descriptive site-level cross-representation alignment/crosswalk. Axis meanings are distributed and nonunique. A52 aligned negatively with all five sea-surface-temperature (SST) summaries (strongest rho = −0.704 for maximum SST), whereas A53 aligned positively with all five (rho = 0.604 for mean SST). Table S3J. Corrected selected-AEF structured null. The 204 rows comprise 68 selected Pfam-AEF pairs across raw-count, total-hit-normalized, and peptide-normalized representations. Each representation used 99,999 intact-site permutations within site phylum-composition strata. Two-sided *p* values equal twice the smaller conditional tail, including ties, and BH correction spans 68 pairs separately within each representation. Table S3K. Corrected selected-AEF seven-specification summary. PF01638-A52 and PF01638-A53 retained direction and selected-family support in all seven required specifications (7/7). PF10988-A36 and PF13411-A18 each passed five of seven specifications (5/7)

Data S1. RbcL alignment and phylogenetic tree, related to Figure 2 and STAR Methods The archive contains the 119-sequence RbcL amino acid alignment and corresponding 119-tip phylogenetic tree in Newick format

Data S2. Analysis programs and scripts, related to STAR Methods The archive contains Python and Google Earth Engine JavaScript programs for protein-family counting, environmental-data extraction, AEF and GEE association analyses, phylogenetic and spatial analyses, visualization, and result-table export

Data S3. HYCOM surface-temperature product-sensitivity analysis, related to STAR Methods and Tables S2I–M The archive contains the analysis programs, source inputs, authenticated outputs, software requirements, provenance manifests, and SHA-256 checksums for the exact-site and equal-weight native-cell HYCOM sensitivity analyses
