# Supplementary material for: Earth-observation variables identify temperature-centered protein-family covariation across macroalgal genomes
Source: iScience. 2026 Oct 7;29(11):117777. doi: 10.1016/j.isci.2026.117777 (PMC13676742; doi:10.1016/j.isci.2026.117777)

iScience, Volume *29*

Supplemental information

Earth-observation variables identify temperature-centered protein-family covariation across macroalgal genomes

Alexandra Mystikou, David R. Nelson, Diana C. El Assal, Ashish K. Jaiswal, Mehar Sultana, Cecilia Rad-Menendez, Noura Al-Mansoori, Sewar T. Elias, Ma-sum Abdul-Hamid, Layanne Nayfeh, Nizar Drou, John A. Burt, David H. Green, and Kourosh Salehi-Ashtiani

Figure S1. Secondary selected-set sensitivity analysis of protein family (Pfam)–AlphaEarth Foundations (AEF) latent-feature associations. Panel A displays rank-association effects for 12 selected pairs. The 12 displayed pairs were selected by an explicit visualization rule: the four priority discovery pairs (PF01638–A53, PF01638–A52, PF10988–A36, and PF13411–A18) plus the eight PF00092 axes with the smallest raw-count unique-site Spearman p values among PF00092–A00 through PF00092–A63 (PF00092–A49, PF00092–A09, PF00092–A31, PF00092–A21, PF00092–A36, PF00092–A50, PF00092–A35, and PF00092–A17). This visualization subset was drawn from the 68-pair selected family and did not define a separate inferential family. The first seven columns are the required gates: unique-site raw counts, total-hit normalization, and peptide normalization; the total-hit quality/phylum site-cluster model; the total-hit Benchmarking Universal Single-Copy Orthologs (BUSCO) ≥50% unique-site analysis; the total-hit three-phylogenetic-eigenvector topology model; and the total-hit structured null. Subsequent columns show additional sensitivity cells and genome-level descriptive estimates. Asterisks indicate Benjamini–Hochberg (BH) $q < 0.05$ across the 68-pair selected family for the indicated method and abundance representation. Panel B shows the total-hit site-mean effect and 2.5th–97.5th percentiles from 99,999 intact-site permutations within site phylum-composition strata. Filled red points indicate corrected conditional-tail BH $q < 0.05$; open blue points indicate $q \geq 0.05$. PF10988–A36 ($p = 0.00304$, $q = 0.05168$) and PF00092–A49 ($p = 0.01626$, $q = 0.221136$) are open. Panel C counts the 12 displayed pairs with nominal, unadjusted $p < 0.05$ at each displayed cell; these descriptive counts are separate from seven-gate candidate retention. PF01638–A52 and PF01638–A53 retained direction and selected-family support across all seven required gates (7/7); PF10988–A36 and PF13411–A18 each passed five of seven (5/7). R denotes raw count, T total-hit normalization, P peptide normalization, Site unique-site Spearman correlation, Qual the quality/phylum site-cluster model, B50 and B70 the BUSCO ≥50% and ≥70% unique-site analyses, P3/P5/P10 the three-, five-, and ten-phylogenetic-eigenvector topology models, Null the structured null, and Geno the genome-level descriptive Spearman correlation. A00–A63 denote unitless latent axes.

Figure S2. Secondary exploratory AEF–Pfam correlation-profile landscape. Average-linkage hierarchical clustering with correlation distance organizes Pfam–AEF Spearman profiles across 126 exact-genome-identifier (ID) records. All 10,707 strict Pfam accessions and 64 axes are displayed as row-wise z scores. The exact- profile audit identified 8,713 unique profiles and 1,994 additional accessions sharing an exact profile; all 10,707 accessions remain in the display. The top bar gives the number of global-BH-supported Pfam pairs per axis, and the right strip gives each Pfam's maximum absolute Spearman coefficient. AEF axes A00–A63 are unitless latent features. Figure S1 reports the selected-set AEF sensitivity analysis. Table S3G–I reports a descriptive site-level cross-representation alignment/crosswalk with named Google Earth Engine variables; axis meanings are distributed and nonunique.

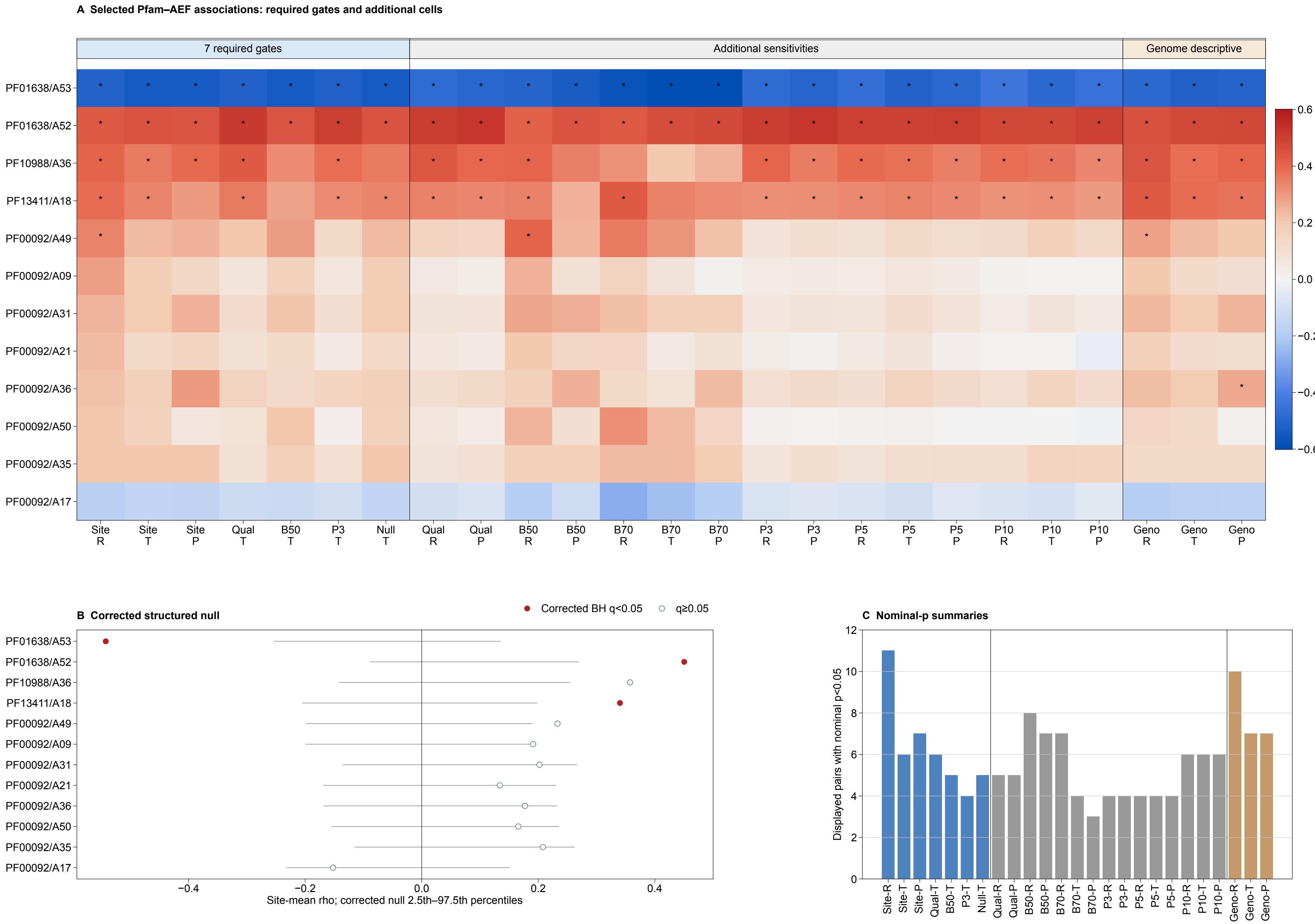
A Selected Pfam–AEF associations: required gates and additional cells
7 required gates
Additional sensitivities
Genome descriptive
PF01638/A53
PF01638/A52
PF10988/A36
PF13411/A18
PF00092/A49
PF00092/A09
PF00092/A31
PF00092/A21
PF00092/A36
PF00092/A50
PF00092/A35
PF00092/A17
Site R
Site T
Site P
Qual T
B50 T
P3 T
Null T
Qual R
Qual P
B50 R
B50 P
B70 R
B70 T
B70 P
P3 R
P3 P
P5 R
P5 T
P5 P
P10 R
P10 T
P10 P
Geno R
Geno T
Geno P
0.6
0.4
0.2
0.0
B Corrected structured null
Corrected BH q<0.05
q≥0.05
PF01638/A53
PF01638/A52
PF10988/A36
PF13411/A18
PF00092/A49
PF00092/A09
PF00092/A31
PF00092/A21
PF00092/A36
PF00092/A50
PF00092/A35
PF00092/A17
−0.4
−0.2
0.0
0.2
0.4
Site-mean rho; corrected null 2.5th–97.5th percentiles
C Nominal-p summaries
Displayed pairs with nominal p<0.05
0
2
4
6
8
10
12
Site-R
Site-T
Site-P
Qual-T
B50-T
P3-T
Null-T
Qual-R
Qual-P
B50-R
B50-P
B70-R
B70-T
B70-P
P3-R
P3-P
P5-R
P5-T
P5-P
P10-R
P10-T
P10-P
Geno-R
Geno-T
Geno-P

Exploratory AEF--Pfam correlation-profile landscape; average linkage, correlation distance

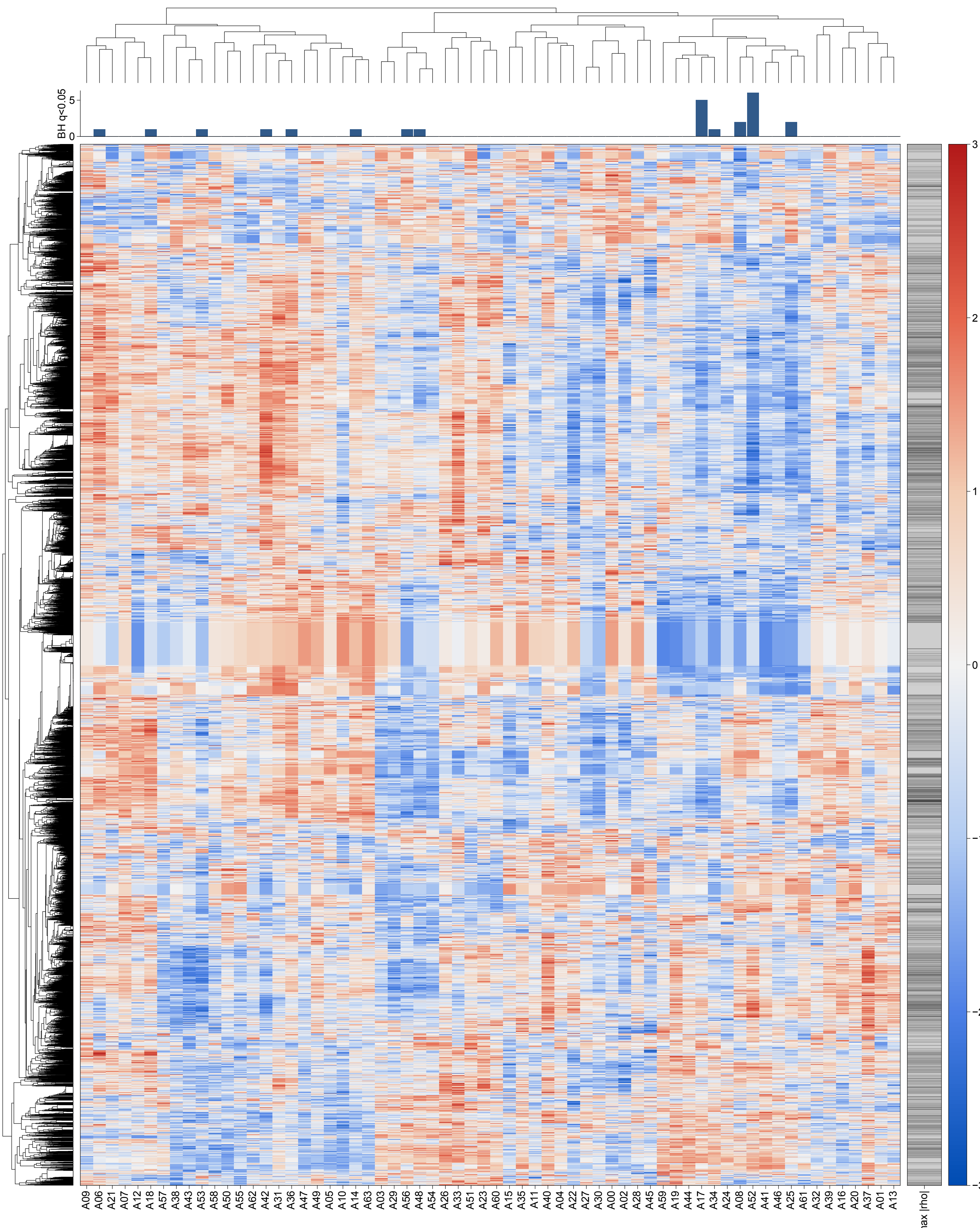

Supplement: Supplementary file 1 [file mmc1.pdf]
